# Methoxylated Chrysin and Quercetin as Potent Stimulators of Melanogenesis

**DOI:** 10.3390/ijms26073281

**Published:** 2025-04-01

**Authors:** Pattara Poungcho, Rita Hairani, Chatchai Chaotham, Wanchai De-Eknamkul, Warinthorn Chavasiri

**Affiliations:** 1 Department of Pharmacognosy and Pharmaceutical Botany, Faculty of Pharmaceutical Sciences, Chulalongkorn University, Bangkok 10330, Thailand; 6271005433@student.chula.ac.th; 2 Center of Excellence in Natural Products Chemistry, Department of Chemistry, Faculty of Science, Chulalongkorn University, Bangkok 10330, Thailand; ritahairani@fmipa.unmul.ac.id; 3 Department of Chemistry, Faculty of Mathematics and Natural Sciences, Mulawarman University, Samarinda 75123, East Kalimantan, Indonesia; 4 Department of Biochemistry and Microbiology, Faculty of Pharmaceutical Sciences, Chulalongkorn University, Bangkok 10330, Thailand; chatchai.c@chula.ac.th

**Keywords:** polymethoxyflavonoids, 5,7-dimethoxychrysin, 3,3′,4′,5,7-pentamethoxyquercetin, chemical synthesis

## Abstract

Polymethoxyflavonoids (PMFs) from plants are known to exhibit melanogenic activity. Very little is known about their structure-activity relationships, and this was the aim of this study. Several series of alkoxy flavonoids were synthesized via semisynthetic and total synthetic pathways. Their structures were identified by NMR analyses, followed by evaluating their potency on the stimulation of melanogenesis using mouse B16F10 and human MNT-1 cells. Among more than twenty methoxylated flavonoids, 5,7-dimethoxychrysin (dimethoxylated chrysin, **F1**) and 3,3′,4′,5,7-pentamethoxyquercetin (pentamethoxylated quercetin, **F21**) appeared to be the most active melanogenic-stimulating compounds in a dose-dependent manner. Both compounds showed no effect on cell viability as determined by MTT assay. The structure-activity relationship study of PMFs revealed that the -OCH_3_ substituent at 5 and 7 positions of A-ring are the most important as melanogenic-stimulating part (e.g., 5,7-dimethoxychrysin, **F1**) followed by at 3′ and 4′ positions of B-ring, and at 3 positions of C-ring (e.g., 3,3′,4′,5,7-pentamethoxyquercetin, **F21**), Therefore, both natural methoxylated flavonoid derivatives of chrysin and quercetin have a potential to be developed further as melanogenic stimulators.

## 1. Introduction

Melanin is a pigment that is essential for human skin, eyes, and hair color. Its formation process (melanogenesis) is triggered by exposure to internal and external stimulators, including ultraviolet (UV) rays, chemicals, and bacteria [1,2]. Tyrosinase is known to be a key enzyme that catalyzes a rate-limiting step in synthesizing melanin [3,4]. Under typical physiological circumstances, this process of pigmentation has a role in protecting human skin from harmful UV radiation and serves a crucial evolutionary role in animal mimicry [5].

The stimulators of melanogenesis through tyrosinase enzyme activity are indispensable agents for cosmetic and pharmaceutical fields, for example, in hair care products and as tanning agents. Additionally, numerous studies have revealed that people with high levels of melanin in their skin have a lower risk of skin cancer [6,7]. On the other hand, pigmentation disorders such as hypopigmentation (low amount of melanin), including piebaldism, pityriasis, and vitiligo, may result from the unbalanced regulation of melanin synthesis [8]. Hence, more melanogenetic research is needed to understand how melanogenesis is controlled in melanocytes [9].

The discovery of melanogenic constituents from natural products and their analogs has become an area of interest due to their low adverse effects with high potential use to improve overall skin conditions [10]. Several research have reported on the melanogenic stimulatory effects of flavonoids, a group of secondary metabolites containing phenolic moiety, including quercetin glycosides and naringenin [11,12,13]. Another unique class of flavonoids is polymethoxyflavonoids (PMFs), which bear two or more methoxy groups on their basic benzo-γ-pyrone skeleton, with a carbonyl group at the C4 position. PMFs have been of noteworthy interest to researchers due to their potent effects on biological activities, such as anti-cancer, anti-neuroinflammatory, and anti-aging [14,15,16,17]. 5-Demethylnobiletin, a natural PMF from citrus fruit peels, stimulates melanogenesis. In vitro and in vivo studies confirm its potential by upregulating key melanogenesis-related genes, interacting with tyrosinase, and exhibiting antioxidative properties. [18,19].

PMFs can be isolated from many natural sources, including *Kaempferia parviflora* Wall. ex. Baker (Zingiberaceae family) which is indigenous to Thailand and known as Krachaidum or black ginger [17]. This plant is popular as a health-promoting herb that has traditionally been used as a folk medicine to lessen blood glucose levels, improve blood flow, and boost vitality [20]. Two major compounds from this plant have been reported, namely 5,7-dimethoxyflavone (DMF) and 5,7,4′-trimethoxyflavone (TMF), with other minor flavonoids [21]. In addition, DMF has been reported to influence melanogenesis which showed significantly increased melanin content and tyrosinase activity without cytotoxicity [17,22].

In this study, several PMFs were synthesized via semisynthetic and total synthetic pathways to investigate the effect of methoxy groups on the DMF scaffold toward melanogenic activity. This was carried out by using chrysin (**1**), the unmethoxylated DMF, as a starting material, followed by its methylation and replacing the methoxy with other alkoxy groups. Furthermore, the correlation between the number of methoxy groups in the B-ring of DMF-based moiety structure toward this assay was also examined. Finally, the active PMFs were evaluated for their effect on melanogenesis in human melanoma MNT-1 cells.

## 2. Results and Discussion

### 2.1. Synthesis of Mono-, Di-, and Poly-Methoxyflavonoid Derivatives

**Scheme 1:**
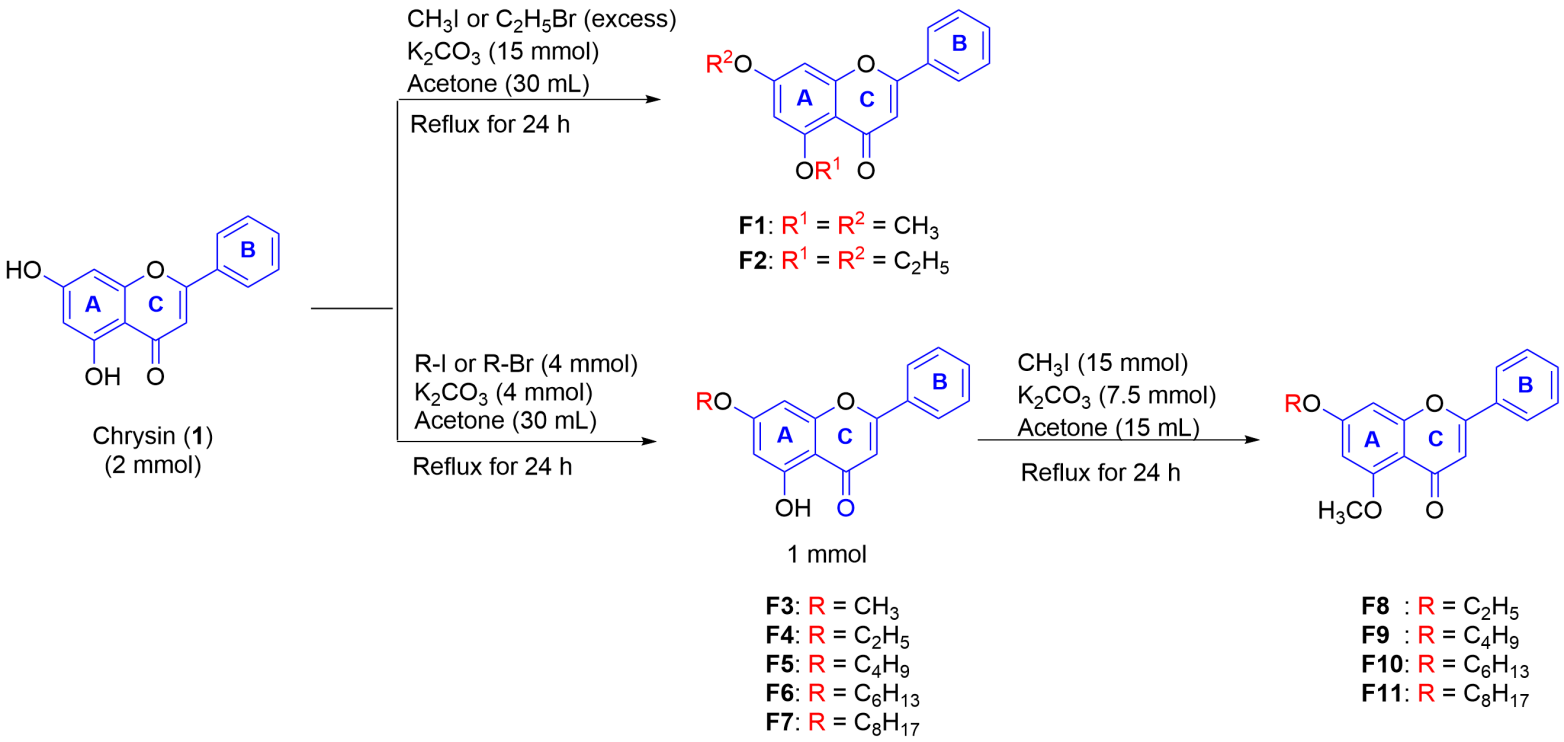
Synthesis of alkoxy chrysin derivatives.

Due to the promising effect of the natural DMF as a stimulator of melanogenesis [17,22] and the intention of our study on the structure-activity relationship of alkoxy and methoxy substituents in the DMF scaffold, chrysin (**1**) was utilized as a starting compound for structural modification and activity determination. Several alkoxy derivatives, including the mono- and dialkoxys of chrysin, were synthesized using the methods described in Scheme 1. All chrysin derivatives were characterized by NMR analysis and/or compared with the previously reported data [23].

As described in Scheme 1, **1** in acetone was reacted with the excess amount of CH_3_I in the presence of K_2_CO_3_ under reflux conditions for 24 h to yield 5,7-dimethoxyflavone (**F1**) as white powder (40%). Additionally, with the aim of observing the effect of carbon atom numbers, 5,7-diethoxyflavone was synthesized (**F2**, white powder, 40%) under similar conditions in the presence of C_2_H_5_Br. Two monoalkoxy chrysin derivatives (**F3** and **F4**) were also synthesized to compare their melanogenic activity with the structures of dialkoxy chrysin. By controlling the ratio amount of RX (alkyl halide), the alkylation could occur at the 7-position in moderate yields to give yellow powder of **F3** (53%) and **F4** (69%). Other monoalkoxy chrysin derivatives (**F5**–**F7**) were prepared and isolated as a pale-yellow powder in moderate to good yields (51–78%). Afterward, to investigate the vital role of the 5-OCH_3_ substituent, the synthesis of several alkoxy chrysin derivatives containing 5-OCH_3_ (**F8**–**F11**) was carried out by methylation of 7-alkoxychrysin derivatives (**F4**–**F7**) using CH_3_I.

**Scheme 2:**
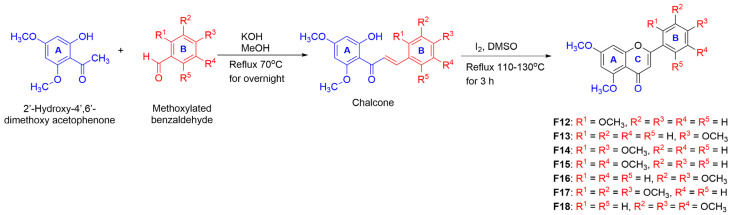
Synthesis of polymethoxyflavonoid derivatives through total synthetic pathway.

Moreover, to study the effect of the -OCH_3_ group in DMF, a number of -OCH_3_ groups were introduced in the B-ring of the DMF-based scaffold. The preparation of this compound series was performed via total synthesis, as shown in Scheme 2. This started from the condensation reaction between 2′-hydroxy-4′,6′-dimethoxyacetophenone and various methoxylated benzaldehyde in the presence of KOH to form chalcones, followed by cyclization using I_2_ in DMSO under reflux to yield **F12**–**F18**. Their structures were analyzed by NMR spectra and identified as known compounds [24,25,26].

**Scheme 3:**
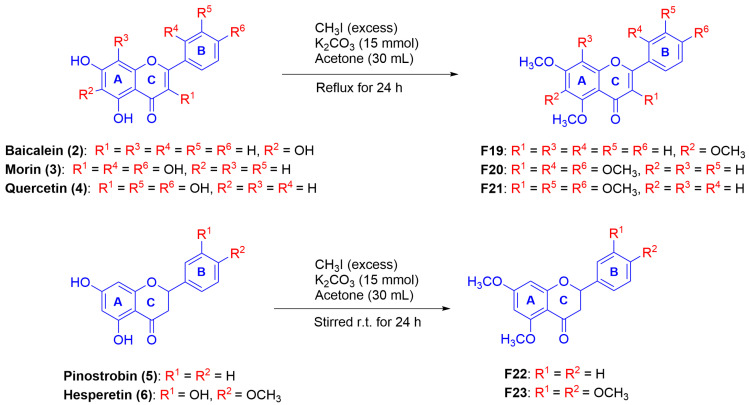
Synthesis of polymethoxyflavonoid derivatives through the semisynthetic pathways.

Additionally, two PMFs were synthesized by methylation of selected natural fla-vonoids (*Scutellaria baicalensis* and *Boesenbergia rotunda* L.) in which all -OH groups were converted into -OCH3 groups using the method described in Scheme 3 to attain derivatives **F19**–**F23** Their structures were elucidated by NMR analysis [27,28]. All these known compounds were then determined for their melanogenetic activity.

### 2.2. The Effects of PMFs on Melanin Production in Mouse B16F10 Melanoma Cells

#### 2.2.1. The Effect of Alkyl Chain Length at 7-Position of 5-OH and 5-OCH_3_ Chrysin (**1**)

Based on the screening of melanin content in B16F10 cells treated with 10 μM of various PMFs for 48 h, forskolin (FSK) at 10 µM was used as a positive control due to its well-established role in stimulating melanogenesis via cAMP activation. Although the precise mechanisms of action of the PMFs have not yet been fully elucidated, forskolin was primarily included to assess the relative potencies of the PMFs rather than to directly compare their efficacy to forskolin. However, we recognize the importance of understanding the mechanisms of action and plan to investigate this aspect in future studies. The results showed that some synthesized compounds, including **F1**, **F2**, **F8**, **F9**, and **F10**, led to an increase in melanin production (Table 1). By modification of chrysin (**1**) in various ways, including (1) methylation at 5- and 7-positions (**F1**), (2) increasing the number of carbon atoms in the dialkoxy substituents (**F2**), (3) the presence of either 5-OH group (**F3** and **F4**) or 5-OCH_3_ substituent (**F1** and **F8**–**F11**), and (4) increasing the carbon length at 7-*O*-position (**F8**–**F11**), many compounds (**F1**, **F2**, **F8**, **F9**, and **F10**) showed significantly mediated higher melanin content than the starting chrysin (**1**) (Table 1). Particularly, the structure of **F1** showed an increase of melanin content almost 2-fold (267.47%). This confirmed that the presence of 5-OCH_3_ in the flavonoid scaffold is important in enhancing melanogenesis, as the replacement of 5-OCH_3_ by 5-OH (**F3** and **F4**) caused a great drop in melanin content. Presumably, this was due to the possible interaction between the -OH substituent with tyrosinase, as reported previously [29,30].

Although the actual cellular target of the treated compounds is still unclear, the tyrosinase enzyme might be postulated to be the most likely one. It has been reported that the -OH groups in flavonoids can be oxidized by tyrosinase to form their corresponding quinones, which leads to the inhibition of the tyrosinase activity [29,30]. Our results showing that the methoxylated flavonoid (**F1**) potentially stimulated the melanogenesis could, therefore, be explained that the replacement of -OH by -OCH_3_ groups in **1** might lessen the interaction of the compound with the tyrosinase enzyme, which favors the melanogenesis process. Alternatively, if the target is not tyrosinase, the synthesized **F1** might act as a compound that is able to stimulate melanocytes by activating the intracellular signaling cascades that lead to increased melanogenetic activity [31,32,33]. In addition, it has been reported that *O*-methylated flavonoids have greater bioavailability due to their better absorption and increased permeability across membranes [34]. Therefore, it is likely that the hydrophobic property of **F1** is the main cause of enhancing the melanin formation. However, the increase in the number of carbon atoms in the di-alkoxy substituents, such as **F2** that contains -OC_2_H_5_ at both 5- and 7-positions, was found to cause significantly melanin declining to 151.66% compared with **F1** (267.47%). Similarly, by increasing the carbon length at only the 7-*O*-position (**F8**–**F11**), the melanin formation also showed to be decreased. This suggested that the higher hydrophobicity caused by the di-alkoxy substituents could limit their diffusion across the phospholipid bilayer. This might be due to the bulkiness of the compounds [35,36].

#### 2.2.2. The Effect of Methoxy Substituents on the B-Ring of 5,7-Dimethoxyflavone (**F1**)

Due to the crucial role of -OCH_3_ on the A-ring of the flavones (Table 1), we continued studying the effect of the -OCH_3_ group attached at different positions on the B-ring of PMFs. A series of derivatives, **F1** and **F12**–**F18,** were synthesized via semisynthetic and total synthetic pathways, and their effect on melanin production was determined. As shown in Table 2, the melanogenic activity of **F1** (with the absence of -OCH_3_ substituent in the B-ring) and of **F12**–**F18** (with various patterns of -OCH_3_ substituents) displayed a variety of values which might depend on both the number and positions of -OCH_3_ substituents. Generally, the addition of -OCH_3_ substituents on the B-ring did not give a significantly beneficial effect on melanogenesis compared with **F1**. This suggested that the presence of the -OCH_3_ group on the B-ring is not essential for the activity, which might be due to the bulkiness and or hydrophobicity properties that limit the effectiveness of the compounds in enhancing melanin production. However, **F12**, **F13**, **F16,** and **F17** still exhibited significant increases in melanin content (173.43–241.47%) compared with the control. The mono-OCH_3_ substituent on the B-ring, **F12** (173.43%) and **F13** (196.33%) showed moderate melanogenetic activity, with the 4′-position being more favored than the 2′-position. The di-OCH_3_ groups at both positions, however, appeared to further decrease the activity, as shown by **F14** (143.41%). Other positions of 2′,5′-substituents were also found to decrease the activity, as shown by **F15** (114.25%), which has two -OCH_3_ substituents at 2′,5′-positions. This suggested that the presence of -OCH_3_ substituents at 2′ and 5′-positions are not crucial for enhancing the melanogenic activity. Interestingly, **F16** containing di- OCH_3_ groups at 3′ and 4′-positions appeared to increase melanin formation by up to 241.47%. It is possible that both -OCH_3_ in *ortho* position could facilitate interaction with the receptors that lead to stimulate the intracellular signaling of the cells. The tri-OCH_3_ at 2′, 3′, and 4′ positions of **F17** (193.74%) showed a slight decrease in activity compared with **F16** (241.47%). Conversely, the tri-OCH_3_ at 3′, 4′, and 5′ positions of **F18** (72.68%) dramatically reduced the melanin content. These results suggested that the number and position of -OCH_3_ groups in the B-ring also influence the activity of melanogenesis [13,18].

#### 2.2.3. The Effect of Fully-Methoxylated Flavonoids on B16F10 Melanin Content

The effect of fully-methoxylated flavonoids on melanin content was further tested using B16F10 cells. The methoxylated flavonoids (**F1**, **F13**, **F19**–**F23**) were synthesized through semisynthesis and total synthesis as described in Section 3.2. As shown in Table 3, **F21** was found to be the most potent compound in enhancing melanogenesis, with a melanin content of 289.71%, followed by **F1** (267.47%). This finding suggested that the 3,3′,4′,5,7-Pentamethoxyflavone has a beneficial effect on melanogenesis. Suggests that the presence of -OCH_3_ at the 3-position of the C-ring is also favored beside the 5- and 7- positions of the A-ring.

By comparing the structures of **F1** and **F13**, the additional -OCH_3_ group on the B-ring of **F13** also influences melanogenesis, but its effect was lower than that of **F1.** A similar result was observed when comparing **F1** and **F19**, where **F19** has an additional -OCH_3_ group at the 6-position on the A-ring. This suggests that the presence of an -OCH_3_ substituent at the 6-position favors the stimulation of melanogenesis.

By comparing **F20** (201.58%) and **F21** (289.71%) containing five -OCH_3_ substituents in both structures, it was found that both pentamethoxylated flavonoids exhibited high melanogenic activity. This suggests that the presence of -OCH_3_ substituent at the 3-position of the C-ring is important in enhancing the activity. **F21,** which exhibited higher activity than **F20,** is likely due to the different position of -OCH_3_ groups on the B-ring, confirming the crucial position of the *ortho* -OCH_3_ groups at 3′,4′-positions rather than *meta* at 2′,4′-positions.

In addition, among the fully-methoxylated flavonoids of **F1**, **F13**, **F19**–**F23**, it appeared that PMFs from the flavanone subclass (**F22** and **F23**) exhibited the lowest enhancing level of B16F10 melanin content. Both flavanones contain no double bond at the C-2 and C-3 positions, suggesting that the presence of a double bond at the C2-C3 position can facilitate the process of melanogenesis [13].

### 2.3. The Effects of PMFs on Melanin Production in Human MNT-1 Cells

**Figure 1:**
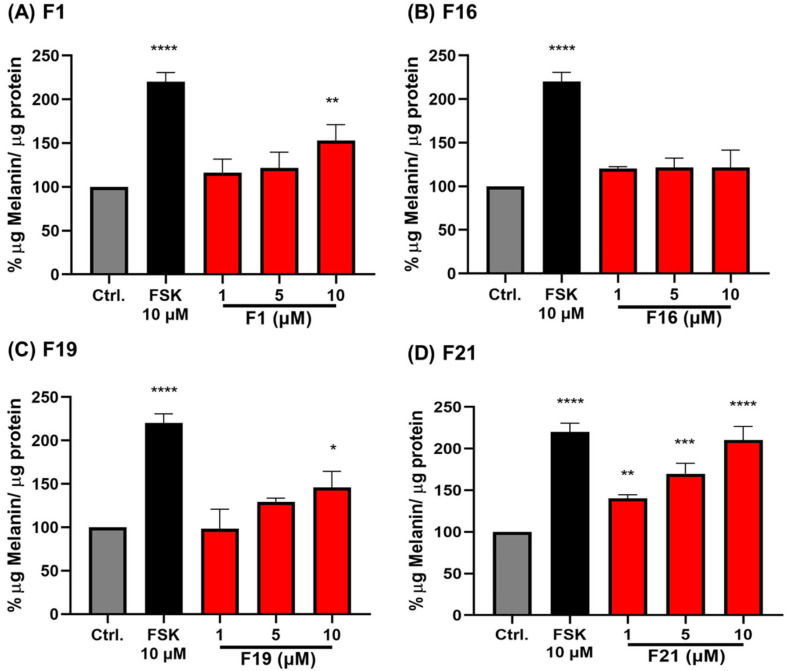
The effect of some selected PMFs on melanin content produced by MNT-1 cells: (**A**) **F1**, (**B**) **F16**, (**C**) **F19,** and (**D**) **F21**. Data represent means ± SD from three independent experiments. * *p* < 0.05, ** *p* < 0.01, *** *p* < 0.001, **** *p* < 0.0001 vs. control groups.

Based on the results shown in Table 3, some PMFs, including **F1**, **F16**, **F19**, and **F21,** exhibited good melanogenic activity in B16F10 mouse melanoma cells and were selected for further study using MNT-1 human melanoma cells. It can be seen that these compounds, except **F16**, clearly stimulated melanogenesis in a dose-dependent manner (Figure 1). The results also showed that **F21** (10 μM) exhibited the strongest melanogenic effect (210.20% μg melanin/μg protein), followed by **F1** (153.07%) and **F19** (146.04%). In contrast, the **F16** treatment did not significantly affect the melanogenesis in MNT-1 cells. Again, **F21** containing five -OCH3 groups appeared to be the most potent melanogenic stimulator for MNT-1 cells. This confirmed that the presence of the -OCH_3_ group at the 3-position in the C-ring is important for the activity. Nevertheless, comparing the melanogenic effect of **F1,** which only contains two -OCH_3_ groups at 5 and 7 positions, and **F19,** which contains three -OCH_3_ groups at 5, 6, and 7 positions, it is obvious that the two -OCH_3_ in the A-ring of **F1** (at 5,6-position) is better than the three -OCH_3_ of **F19** (at 5,6,7-position). It might be that the **F19** creates steric hindrance, causing the decline of the melanogenic effect.

Notably, the effect of some selected PMFs with potent melanogenic activity, including **F1**, **F16**, and **F19**–**F21,** on the viability of B16F10 and MNT-1 cells was assessed. This was carried out by MTT assay method in which cells were treated with various concentrations of the compounds for 48 h. The results showed that all the selected potent compounds showed no cytotoxicity at any concentrations from 1 to 10 μM on B16F10 (Figure S1) and MNT-1 (Figure S2).

### 2.4. Structure-Activity Relationship of PMFs on Melanogenic Enhancing Activity

**Figure 2:**
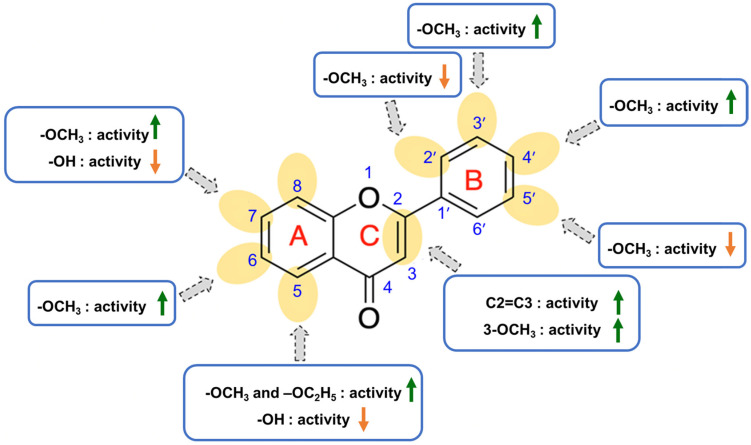
Structure-activity relationship of PMFs on B16F10 melanin content.

Based on the findings, the structure-activity relationship of PMF derivatives could be rationalized and illustrated in Figure 2. First, the presence of -OCH_3,_ as well as its number and position, could influence the stimulation of melanogenesis. Second, the increase in the number of carbon atoms of 7-*O*-alkyl of 5-OCH_3_ chrysin did not increase the production of melanin more than the presence of 7-OCH_3_ in B16F10 cells. Finally, the position and the presence of the substituents that have steric hindrance tend to decline the activity.

## 3. Materials and Methods

### 3.1. Materials

Two plant materials were used in this work, including the rhizome of *Boesenbergia rotunda,* which was purchased from the herbal drug store in Bangkok-Thailand, and the rhizome of *Scutellaria baicalensis* from the herbal drug store in China. Chrysin, morin, and quercetin were purchased from the Tokyo Chemical Industry Company (Tokyo Chemical Industry, Tokyo, Japan) and used without further purification. The synthetic reagents, including 2′-hydroxy-4′,6′-dimethocyacetophenone, KOH, hesperetin, and others, were purchased from Merck Chemical Company (Darmstadt, Germany) or otherwise stated. All solvents used in this study were purified by standard methods, except for those which were reagent grades. The progress of the reaction was monitored by Thin Layer Chromatography (TLC) using TLC silica gel 60 F254 Merck. The purification of semisynthetic compounds was performed by column chromatography using silica gel (70–230 mesh) of SiliaFlash^®^ G60 (SiliCycle, Québec City, QC, Canada). 1H NMR (500 MHz) and 13C NMR (125 MHz) spectra were recorded with a JEOL spectrometer (JNM-ECZ500R/S1, JEOL, Tokyo, Japan), and chemical shifts were recorded in parts per million (ppm) and coupling constants (J) were given in Hz. The LC-QTOF-MS/MS analysis was performed on an Agilent HPLC 1260 series coupled with a QTOF 6540 UHD accurate mass (Agilent Technologies, Waldbronn, Germany).

### 3.2. Methods

#### 3.2.1. Isolation of Natural Flavonoids

##### Isolation of Baicalein from *Scutellaria baicalensis* Roots

The air-dried powdered of *Scutellaria baicalensis* roots (0.4 kg) were refluxed in MeOH for 6 h, then concentrated by a Buchi rotary evaporator to give MeOH extract. This action was performed twice. The MeOH extract was then successively partitioned with hexane, CH_2_Cl_2_, EtOH, and MeOH-H_2_O. A Buchi rotary evaporator was used to concentrate the CH_2_Cl_2_ filtrate to get the CH_2_Cl_2_ fraction. Following a silica gel column chromatography procedure, approximately 5 g of the CH_2_Cl_2_ fraction was initially eluted with hexane-EtOAc and EtOAc-MeOH by increasing polarity to yield five fractions (1–5). From fraction 3, baicalein (**F2**) was isolated as a yellow powder (0.1%).

Baicalein (**F2**): yellow solid (0.1%), ^1^H NMR (DMSO-*d_6_*) δ (ppm) 8.06 (dd, *J* = 8.0, 1.5 Hz, 2H), 7.58 (m, 3H), 6.93 (s, 1H), and 6.63 (s, 1H). ^13^C NMR (DMSO-*d_6_*) δ (ppm) 182.2, 163.0, 153.7, 149.9, 147.0, 131.9, 131.0, 129.4, 129.2, 126.4, 104.5, 104.3, and 94.1.

##### Isolation of Pinostrobin from *Boesenbergia rotunda* L. Rhizomes

One kilogram of dried and powdered *Boesenbergia rotunda* L. rhizomes was extracted by soaking in MeOH for 3 days at room temperature, filtered, and the solvent was removed by a Buchi rotary evaporator. The process was carried out 3 times. After that, this MeOH extract was subjected to silica gel column chromatography, which was initially eluted with hexane-EtOAc and EtOAc-MeOH by increasing polarity. The process gave seven fractions. Pinostrobin (**F5**) was obtained as a colorless crystal (64 g, 46%).

Pinocembrin (**F5**): pale-yellow solid (16%), ^1^H NMR (Acetone-*d_6_*) δ (ppm) 12.16 (s, 5-OH), 9.76 (s, 7-OH), 7.57 (dd, *J* = 7.0, 1.5 Hz, 2H), 7.42 (m, 3H), 6.00 (d, *J* = 2.5 Hz, 1H), 5.97 (d, *J* = 2.0 Hz, 1H), 5.57 (dd, *J* = 13.0, 3.5 Hz, 1H), 3.17 (dd, *J* = 17.0, 12.5 Hz, 1H), and 2.81 (dd, *J* = 17.0, 3.0 Hz, 1H); ^13^C NMR (Acetone-*d6*) δ (ppm) 196.8, 167.4, 165.2, 164.1, 140.0, 129.4, 127.3, 103.2, 96.9, 95.9, 79.9, and 43.5.

#### 3.2.2. General Procedure for the Synthesis of Flavonoid Derivatives

##### Synthesis of Chrysin Derivatives (**F1**–**F15**)

To attain di-alkoxy chrysin derivatives (**F1** and **F2**), the presence of CH_3_I or C_2_H_5_Br in excess amount was added into a solution of **1** (2 mmol) in 30 mL acetone in the presence of anhydrous K_2_CO_3_ (5 mmol), then followed by refluxing the mixture for 24 h. Afterward, K_2_CO_3_ was removed by filtration. The filtrate was concentrated by a rotary evaporator, then purified by a silica gel column and eluted with hexane:EtOAc from 20% Hexane/EtOAc until 100% EtOAc.

Derivatives **F3**–**F7** containing 5-OH by varying the alkyl chain in the 7-*O* position of chrysin were synthesized by treating **1** (1 mmol) with alkyl bromide or alkyl iodide (2 mmol) in the presence of anhydrous K_2_CO_3_ (5 mmol) in acetone (15 mL), then followed by refluxing the mixture for 24 h. Then, K_2_CO_3_ was removed by filtration. The filtrate was concentrated by a rotary evaporator, then purified by a silica gel column and eluted with hexane:EtOAc (4:1) to give **F3**–**F7**.

Selected 1 mmol of 7-*O*-ether chrysin derivatives (**F4**–**F7**) were further methylated with CH_3_I (2 mmol) in the presence of anhydrous K_2_CO_3_ (5 mmol) in acetone (15 mL), then followed by refluxing the mixture for 24 h and filtration. The filtrate was then purified by a silica gel column and eluted with hexane:EtOAc from 20% Hexane/EtOAc until 100% EtOAc to provide **F8**–**F11**.

5,7-Dimethoxyflavone (**F1**): white powder (40%). ^1^H NMR (CDCl_3_) δ (ppm) 7.87 (dd, *J* = 7.0, 2.0 Hz, 2H), 7.49 (m, 3H), 6.68 (s, 1H), 6.57 (d, *J* = 2.0 Hz, 1H), 6.37 (d, *J* = 3.5 Hz, 1H), 3.95 (s, 3H), and 3.91 (s, 3H). ^13^C NMR (CDCl_3_) δ (ppm) 177.8, 164.2, 161.0, 160.9, 160.1, 131.6, 131.4, 129.1, 126.1, 109.4, 109.1, 96.3, 93.0, 56.6, and 55.9.

5,7-Diethoxyflavone (**F2**): white powder (40%). ^1^H NMR (acetone-*d_6_*) δ (ppm) 8.00 (dd, *J* = 6.5, 2.5 Hz, 2H), 7.57 (m, 3H), 6.74 (s, 1H), 6.60 (d, *J* = 2.0 Hz, 1H), 6.38 (d, *J* = 2.3 Hz, 1H), 6.44 (s, 1H), 4.19 (m, 2H), 4.12 (m, 2H), and 1.42 (m, 6H). ^13^C NMR (acetone-*d_6_*) δ (ppm) 176.4, 164.2, 161.0, 160.7, 160.6, 132.5, 131.9, 129.8, 126.7, 109.9, 109.3, 98.1, 94.3, 65.4, 64.8, and 14.8.

7-Methoxy-5-hydroxyflavone (**F3**): yellow powder (53%). ^1^H NMR (CDCl_3_) δ (ppm) 7.87 (dd, *J* = 8.0, 1.5 Hz, 2H), 7.52 (m, 3H), 6.65 (s, 1H), 6.48 (d, *J* = 2.0 Hz, 1H), 6.36 (d, *J* = 2.0 Hz, 1H), and 3.87 (s, 3H). ^13^C NMR (CDCl_3_) δ (ppm) 182.6, 165.7, 164.1, 162.3, 157.9, 132.0, 131.5, 129.2, 126.4, 105.9, 105.8, 98.3, 92.8, and 55.9.

7-Ethoxy-5-hydroxyflavone (**F4**): yellow powder (69%). ^1^H NMR (CDCl_3_) δ (ppm) 7.88 (dd, *J* = 8.0, 1.5 Hz, 2H), 7.52 (m, 3H), 6.66 (s, 1H), 6.48 (d, *J* = 2.5 Hz, 1H), 6.36 (d, *J* = 2.0 Hz, 1H), 4.11 (q, *J* = 7.0 Hz, 2H), and 1.45 (t, *J* = 7.0 Hz, 3H). ^13^C NMR (CDCl_3_) δ (ppm) 182.6, 165.2, 164.1, 162.3, 157.9, 132.0, 131.5, 129.2, 126.4, 105.9, 105.7, 98.7, 93.2, 64.4, and 14.7.

7-Butoxy-5-hydroxyflavone (**F5**): pale yellow powder (58%). ^1^H NMR (CDCl_3_) δ (ppm) 7.88 (dd, *J* = 8.0, 1.5 Hz, 2H), 7.53 (m, 3H), 6.66 (s, 1H), 6.49 (d, *J* = 2.0 Hz, 1H), 6.36 (d, *J* = 2.0 Hz, 1H), 4.04 (t, *J* = 7.0 Hz, 2H), 1.80 (m, 2H), 1.51 (m, 2H), and 0.99 (t, *J* = 7.0 Hz, 3H). ^13^C NMR (CDCl_3_) δ (ppm) 182.6, 165.4, 164.1, 162.2, 157.9, 132.0, 129.2, 126.4, 106.0, 105.7, 98.8, 93.2, 68.5, 31.1, 19.3, and 13.9.

7-Hexyloxy-5-hydroxyflavone (**F6**): pale yellow powder (76%). ^1^H NMR (CDCl_3_) δ (ppm) 7.87 (dd, *J* = 8.0, 1.5 Hz, 2H), 7.52 (m, 3H), 6.65 (s, 1H), 6.48 (d, *J* = 2.5 Hz, 1H), 6.35 (d, *J* = 2.0 Hz, 1H), 4.02 (t, *J* = 6.5 Hz, 2H), 1.81 (m, 2H), 1.46 (m, 2H), 1.35 (m, 4H), and 0.92 (t, *J* = 7.0 Hz, 3H). ^13^C NMR (CDCl_3_) δ (ppm) 182.6, 165.4, 164.0, 162.2, 157.9, 131.9, 131.5, 129.2, 126.4, 105.9, 105.7, 98.7, 93.2, 68.8, 31.6, 29.0, 25.7, 22.7, and 14.2.

5-Hydroxy-7-octyloxyflavone (**F7**): pale yellow powder (51%). ^1^H NMR (CDCl_3_) δ (ppm) 7.87 (dd, *J* = 8.0, 1.5 Hz, 2H), 7.53 (m, 3H), 6.66 (s, 1H), 6.49 (d, *J* = 2.0 Hz, 1H), 6.36 (d, *J* = 2.5 Hz, 1H), 4.02 (t, *J* = 6.5 Hz, 2H), 1.81 (m, 2H), 1.46 (m, 2H), 1.32 (m, 8H), and 0.89 (t, *J* = 7.0 Hz, 3H). ^13^C NMR (CDCl_3_) δ (ppm) 182.6, 165.3, 164.0, 162.2, 157.9, 131.9, 131.5, 129.2, 126.4, 106.0, 105.7, 98.8, 93.2, 68.9, 31.9, 29.4, 29.3, 29.1, 26.1, 22.8, and 14.2.

7-Ethoxy-5-methoxyflavone (**F8**): yellow powder (69%). ^1^H NMR (DMSO-*d6*) δ (ppm) 8.02 (dd, *J* = 7.5, 1.5 Hz, 2H), 7.55 (m, 3H), 6.82 (d, *J* = 2.0 Hz, 1H), 6.75 (s, 1H), 6.47 (d, *J* = 2.5 Hz, 1H), 4.16 (q, *J* = 7.5 Hz, 2H), 3.82 (s, 3H), and 1.37 (t, *J* = 7.0 Hz, 3H). ^13^C NMR (DMSO-*d6*) δ (ppm) 175.7, 163.1, 160.3, 159.6, 159.2, 131.4, 130.9, 129.1, 125.9, 108.3, 108.2, 96.6, 93.7, 64.1, 56.1, and 14.4.

7-Butoxy-5-methoxyflavone (**F9**): pale yellow powder (58%). ^1^H NMR (DMSO-*d6*) δ (ppm) 8.03 (dd, *J* = 7.0, 1.5 Hz, 2H), 7.55 (m, 3H), 6.85 (d, *J* = 3.0 Hz, 1H), 6.76 (s, 1H), 6.47 (d, *J* = 2.0 Hz, 1H), 4.10 (t, *J* = 6.5 Hz, 2H), 3.82 (s, 3H), 1.73 (m, 2H), 1.45 (m, 2H), and 0.95 (t, *J* = 7.5 Hz, 3H). ^13^C NMR (DMSO-*d6*) δ (ppm) 175.7, 163.2, 160.3, 159.5, 159.2, 131.4, 130.9, 129.1, 125.9, 108.3, 108.2, 96.6, 93.7, 68.1, 56.1, 30.5, 18.7, and 13.7.

7-Hexyloxy-5-methoxyflavone (**F10**): pale yellow powder (76%). ^1^H NMR (CDCl_3_) δ (ppm) 7.86 (dd, *J* = 7.0, 2.0 Hz, 2H), 7.49 (m, 3H), 6.68 (s, 1H), 6.55 (d, *J* = 2.0 Hz, 1H), 6.37 (d, *J* = 2.5 Hz, 1H), 4.05 (t, *J* = 6.5 Hz, 2H), 3.95 (s, 3H), 1.82 (m, 2H), 1.49 (m, 2H), 1.36 (m, 4H), and 0.92 (t, *J* = 7.5 Hz, 3H). ^13^C NMR (CDCl_3_) δ (ppm) 177.8, 163.8, 161.0, 160.8, 160.0, 131.7, 131.3, 129.1, 126.1, 109.2, 109.1, 96.7, 93.4, 68.8, 56.5, 31.6, 29.1, 25.8, 22.7, and 14.1

5-Methoxy-7-octyloxyflavone (**F11**): pale yellow powder (51%). ^1^H NMR (CDCl_3_) δ (ppm) 7.86 (dd, *J* = 7.5, 2.05 Hz, 2H), 7.49 (m, 3H), 6.55 (d, *J* = 2.0 Hz, 1H), 6.37 (d, *J* = 2.5 Hz, 1H), 4.05 (t, *J* = 6.5 Hz, 2H), 3.95 (s, 3H), 1.82 (m, 2H), 1.48 (m, 2H), 1.33 (m, 8H), and 0.89 (t, *J* = 6.5 Hz, 3H). ^13^C NMR (CDCl_3_) δ (ppm) 177.8, 163.8, 161.0, 160.1, 131.7, 131.3, 129.1, 126.1, 109.2, 109.1, 96.7, 93.4, 68.8, 56.6, 31.9, 29.4, 29.3, 29.1, 26.1, 22.8, and 14.2.

##### Synthesis of PMFs **F12–F18**

To a round-bottom flask containing 2′-hydroxy-4′,6′-dimethoxyacetophenone (1 mmol), methoxylated benzaldehyde (1.1 mmol), and 50% aqueous KOH (2 mL) in 15 mL of MeOH, the mixture was then vigorously stirred at 70 °C overnight.The reaction mixture was poured into ice/cold water and extracted with CH_2_Cl_2_. Afterward, the organic layer was concentrated and purified by a silica gel column to give 2-hydroxychalcone.

To a solution of 2-hydroxychalcone (1 mmol) in DMSO was added I_2_. The mixture was refluxed for 3 h. Subsequently, the mixture was poured into water and extracted with EtOAc. The organic layer was washed with brine, and the solvent was evaporated. The residue was then purified by a silica gel column.

2′,5,7-Trimethoxyflavone (**F12**): pale yellow solid (88%). ^1^H NMR (CDCl_3_) δ (ppm) 7.87 (d, *J* = 7.9 Hz, 2H), 7.45 (t, *J*= 7.6 Hz, 2H), 7.02 (s, 1H), 6.54 (d, *J =* 2.3 Hz, 1H), 6.36 (d, *J =* 2.3 Hz, 1H), 4.01-3.85 (m, 9H).

4′,5,7-Trimethoxyflavone (**F13**): white powder (83%). ^1^H NMR (acetone-*d6*) δ (ppm) 8.00 (dd, *J* = 6.5, 2.5 Hz, 2H), 7.57 (m, 3H), 6.74 (s, 1H), 6.60 (d, *J* = 2.0 Hz, 1H), 6.38 (d, *J* = 2.3 Hz, 1H), 6.44 (s, 1H), 4.19 (m, 2H), 4.12 (m, 2H), and 1.42 (m, 6H). ^13^C NMR (acetone-*d6*) δ (ppm) 176.4, 164.2, 161.0, 160.7, 160.6, 132.5, 131.9, 129.8, 126.7, 109.9, 109.3, 98.1, 94.3, 65.4, 64.8, and 14.8.

2′,4′,5,7-Tetramethoxyflavone (**F14**): pale brown solid (92%). ^1^H NMR (CDCl_3_) δ (ppm) 7.85 (d, *J* = 8.7 Hz, 1H), 6.61 (dd, *J*= 8.8, 2.4 Hz, 2H), 6.53 (d, *J =* 2.6 Hz, 2H), 6.36 (d, *J =* 2.3 Hz, 1H), 3.91 (dd, *J* = 19.0, 8.5 Hz, 12H).

2′,5,5′,7-Tetramethoxyflavone (**F15**): pale brown solid (87%). ^1^H NMR (CDCl_3_) δ (ppm) 7.44 (d, *J* = 3.2 Hz, 2H), 6.99 (m, 2H), 6.57 (d, *J =* 2.3 Hz, 1H), 6.38 (d, *J =* 2.3 Hz, 1H), 4.19 − 3.75 (m, 12H).

3′,4′,5,7-Tetramethoxyflavone (**F16**): pale yellow powder (80%). ^1^H NMR (CDCl_3_) δ (ppm) 7.64 (dd, *J* = 2.5, 2.5 Hz, 1H), 7.52 (d, *J* = 2 Hz, 1H), 6.87 (d, *J* = 2.5 Hz, 1H), 6.77 (s, 1H), 6.50 (d, *J* = 2.0 Hz, 1H), 3.90 (s, 3H), 3.88 (s, 3H), 3.84 (s, 3H), and 3.82 (s, 3H). ^13^C NMR (CDCl_3_) δ (ppm) 175.9, 163.7, 160.3, 159.8, 159.3, 151.6, 149.1, 123.2, 119.4, 111.7, 109.1, 108.3, 107.1, 96.3, 93.5, 56.1, 56.1, 55.9, and 55.8.

2′,3′,4′,5,7-Pentamethoxyflavone (**F17**): pale brown solid (91%). ^1^H NMR (CDCl_3_) δ (ppm) 7.48 (d, *J* = 8.9 Hz, 1H), 6.77 (s, 1H), 6.71 (d, *J =* 8.9 Hz, 1H), 6.49 (d, *J =* 2.4 Hz, 1H), 6.35 (d, *J* = 15.5 Hz, 1H), 4.12 − 3.70 (m, 15H).

3′,4′,5,5′,7-Pentamethoxyflavone (**F18**): brown solid (86%). ^1^H NMR (CDCl_3_) δ (ppm) 7.10 (s, 2H), 6.90 (s, 1H), 6.61 (d, *J =* 2.3 Hz, 1H), 6.40 (d, *J =* 2.2 Hz, 1H), 4.17 − 3.77 (m, 15H).

##### Synthesis of PMFs **F19–F23**

To obtain semisynthetic compounds, **F19**–**F23** was performed by a protocol described for synthesizing **F1**, in which the starting materials utilized were baicalein, morin, quercetin, pinostrobin, and hesperetin, respectively.

5,6,7-Trimethoxyflavone (**F19**): white powder (78%). ^1^H NMR (CDCl_3_) δ (ppm) 7.89 (m, 2H), 7.51 (m, 3H), 6.83 (s, 1H), 6.76 (s, 1H), 3.99 (s, 6H), and 3.92 (s, 3H). ^13^C NMR (CDCl_3_) δ (ppm) 177.5, 161.6, 158.1, 154.8, 152.7, 140.6, 131.6, 129.1, 126.2, 112.9, 108.3, 96.4, 62.4, 61.7, and 56.5.

2′,3,4′,5,7-Pentamethoxyflavone (**F20**): white powder (53%). ^1^H NMR (DMSO-*d6*) δ (ppm) 7.64 (m, 2H), 7.12 (d, *J* = 10 Hz), 6.80 (s, 1H),6.47 (s, 1H), 3.88 (s, 3H), 3.85 (s, 3H), 3.84 (s, 3H), 3.83 (s, 3H), and 3.74 (s, 3H).

3,3′,4′,5,7-Pentamethoxyflavone (**F21**): white powder (69%). ^1^H NMR (CDCl_3_) δ (ppm) 7.70 (m, 2H), 6.97 (d, *J* = 7.5 Hz, 1H), 6.50 (d, *J* = 2.5 Hz, 1H),6.34 (d, *J* = 2.0 Hz, 1H), 3.96 (s, 9H), 3.90 (s, 3H), and 3.87 (s, 3H). ^13^C NMR (CDCl_3_) δ (ppm) 174.2, 164.0, 161.1, 158.9, 152.7, 150.9, 148.8, 141.3, 123.5, 121.8, 111.4, 110.9, 109.6, 95.9, 92.6, 60.1, 56.5, 56.2, 56.1, and 55.9.

5,7-Dimethoxyflavanone (**F22**): white powder (58%). ^1^H NMR (CDCl_3_) δ (ppm) 7.89 (d, *J* = 20 Hz, 1H), 7.78 (d, *J* = 15 Hz, 1H), 7.62 (d, *J* = 5 Hz, 2H), 7.42 (m, 5H), 6.01 (s, 1H), 3.96 (s, 3H), and 3.91 (s, 3H).

3′,4′,5,7-Tetramethoxyflavanone (**F23**): white powder (76%). ^1^H NMR (CDCl_3_) δ (ppm) 6.99 (d, *J* = 10 Hz, 2H), 6.89 (m, 1H), 6.15 (d, J = 2.3 Hz, 1H), 6.09 (d, *J* = 2.3 Hz, 1H), 5.34 (dd, *J* = 2.9, 2.6 Hz, 1H), 3.92 (s, 3H), 3.89 (s, 6H), 3.82 (s, 3H), 3.04 (dd, *J* = 13.45, 13.15 Hz, 1H), and 2.77 (dd, *J* = 2.9, 2.9 Hz, 1H). ^13^C NMR (CDCl_3_) δ (ppm) 189.6, 166.1, 165.1, 162.4, 149.5, 149.3, 131.3, 119.0, 111.2, 109.4, 106.1, 93.7, 93.3, 79.3, 56.3, 56.1, 55.7, and 55.6.

#### 3.2.3. Melanogenesis Activity

##### Cell Culture

Mouse melanoma B16F10 cells were purchased from ATCC (American Type Culture Collection, Manassas, VA, USA) and cultured in high glucose Dulbecco’s Modified Eagle’s Medium (DMEM), without glutamine and phenol red (Gibco, Waltham, MA, USA), containing 10% fetal bovine serum (Gibco, Waltham, MA, USA), 1% antibiotic-antimycotic (Gibco, Waltham, MA, USA), and 1% glutamax (Gibco, Waltham, MA, USA). Human melanoma MNT-1 cells were purchased from ATCC (Manassas, VA, USA) and cultured in DMEM-high glucose containing 20% fetal bovine serum, 10% of AIM-V medium, 1% antibiotic-antimycotic, 1% glutamax, and 1% sodium pyruvate. Both B16F10 and MNT-1 cells were cultivated in the standard condition, 37 °C, 5% CO_2_ until they reached 70–80% confluence for the next experiments.

##### Cell Viability Assay

To determine the non-toxic concentrations of synthesized compounds, the viability of cells following treatment with extracts is determined by MTT assay. This method is based on the reduction of 3-(4, 5-dimethylthiazol-2-yl)-2, 5-diphenyl tetrazolium bromide (MTT) (Thermo Fisher Scientific, Waltham, MA, USA) to formazan by mitochondrial enzymes in viable cells. The quantity of formed formazan is proportional to the number of viable cells and can be measured spectrophotometrically. Briefly, B16F10 and MNT-1 cells were seeded at a density of 5 × 10^3^ cells/well in a 96-well plate and incubated to adhere overnight. After treatment with synthesized compounds at different concentrations for 48 h, the cells were treated with 5 mg/mL MTT for 4 h at 37 °C in the incubator (NuAire, Nu8500E, Plymouth, MN, USA). Then, the supernatant was removed, and DMSO was to dissolve the formazan product. The intensity was measured at 570 nm using a microplate reader (Perkin Elmer, Victor3, Waltham, MA, USA).

##### Measurement of Melanin Content

B16F10 cells were seeded at a density of 5 × 10^3^ cells/well in a 96-well plate in DMEM without phenol red and incubated to adhere overnight. After 24 h, the B16F10 cells were treated with the synthesized compounds at non-toxic concentrations (10 μM) and incubated for 48 h at optimum conditions for melanin production. The melanin production was evaluated through the microplate reader at a wavelength of 405 nm. DMEM and Forskolin (10 μM) served as a negative and positive control, respectively.

MNT-1 cells were seeded in 6 well plates at density 1 × 10^5^ cell/well/2 mL, and cells were incubated overnight. Then, the cells were treated with a non-toxic concentration of the synthesized compound for 72 h. Afterward, the medium was removed and washed with DPBS twice. Cells were collected in 10% RIPA lysis buffer by using a cell scarper. Then, cells were incubated in lysis buffer for 45 min and centrifuged at 12,000 rpm and 4 °C for 15 min; then, the supernatant was collected to determine the amount of protein using a BCA assay (Thermo Fisher Scientific, Waltham, MA, USA). The cellular pellets were diluted in 1N NaOH with 10% DMSO and were further incubated at 80 °C for 3 h. After centrifugation at 12,000 rpm and 25 °C for 10 min, melanin content was measured, and the absorbance was at 405 nm. The amount of melanin was compared with the melanin standard curve, which is shown in µg/mL. The melanin content was normalized to the amount of protein. Therefore, the melanin content was represented as µg melanin/µg protein.

##### Statistical Analysis

All the experiments were performed in triplicate, and the results were expressed as the mean ± standard error of the mean (SEM). Statistical analysis was performed using one-way ANOVA using GraphPad Prism 9.0 software; a *p*-value < 0.05 was considered statistically significant.

## 4. Conclusions

Several PMFs exhibited promising melanogenic effects with different activities on B16F10 mouse melanoma cells, including **F1**, **F2**, **F8**, **F11**, **F13**, and **F16**–**F21**. The position of -OCH_3_ groups in the flavonoid scaffold played a vital role in melanogenic stimulation. **F1** and **F21** exhibited strong melanogenic-stimulating effects on human MNT-1 melanoma cells without any cytotoxic effect. **F1** and **F21** are natural PMFs known as derivatives of chrysin (5,7-dimethoxychrysin) and quercetin (3,3′,4′,5,7-pentamethoxy quercetin). These findings suggest that **F1** and **F21** have the potential to be developed further as melanogenic stimulators. However, further studies on their mechanism of action should be carried out to understand how the compounds affect the process of melanogenesis.

## Figures and Tables

**Table 1:** Effect of alkyl chain length at 7-position of 5-OH and 5-OCH_3_ on B16F10 melanin content.

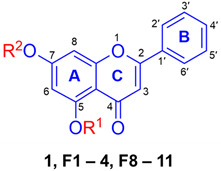			
Compound	R^1^	R^2^	% Melanin Content
Chrysin (**1**)	H	H	141.00 ± 1.15
**F1**	CH_3_	CH_3_	267.47 ± 1.30 ****
**F2**	C_2_H_5_	C_2_H_5_	151.66 ± 2.81 *
**F3**	H	CH_3_	113.61 ± 1.69
**F4**	H	C_2_H_5_	138.44 ± 1.18
**F8**	CH_3_	C_2_H_5_	163.39 ± 1.52 **
**F9**	CH_3_	C_4_H_9_	179.15 ± 0.45 ****
**F10**	CH_3_	C_6_H_13_	160.19 ± 2.75 **
**F11**	CH_3_	C_8_H_17_	88.77 ± 0.57
Forskolin (Positive control)			291.94 ± 0.82 ****
No compound added (Control)			100 ± 00

Significant differences compared to untreated control cells are expressed by * *p* < 0.05, ** *p* < 0.01, and **** *p* < 0.0001.

**Table 2:** Effect of the number of methoxy substituents on B-ring of 5,7-dimethoxyflavone (**F1**) on melanogenic activity.

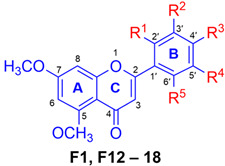						
Compounds	R^1^	R^2^	R^3^	R^4^	R^5^	% Melanin Content
**F1**	H	H	H	H	H	267.47 ± 1.30 ****
**F12**	OCH_3_	H	H	H	H	173.43 ± 2.14 ***
**F13**	H	H	OCH_3_	H	H	196.33 ± 1.82 ****
**F14**	OCH_3_	H	OCH_3_	H	H	143.41 ± 1.68
**F15**	OCH_3_	H	H	OCH_3_	H	114.25 ± 2.93
**F16**	H	OCH_3_	OCH_3_	H	H	241.47 ± 3.37 ****
**F17**	OCH_3_	OCH_3_	OCH_3_	H	H	193.74 ± 3.69 ****
**F18**	H	OCH_3_	OCH_3_	OCH_3_	H	72.68 ± 0.97
Forskolin (positive control)						291.94 ± 0.82 ****
No compound added (control)						100 ± 00

Significant differences compared to untreated control cells are expressed by *** *p* < 0.001, and **** *p* < 0.0001.

**Table 3:** Effect of various methoxylated flavonoids on melanogenesis activity.

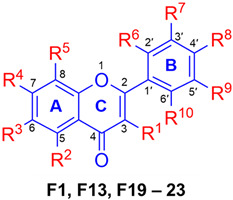											
Compounds	R^1^	R^2^	R^3^	R^4^	R^5^	R^6^	R^7^	R^8^	R^9^	R^10^	% Melanin Content
**F1**	H	OCH_3_	H	OCH_3_	H	H	H	H	H	H	267.47 ± 1.30 ****
**F13**	H	OCH_3_	H	OCH_3_	H	H	H	OCH_3_	H	H	196.33 ± 1.82 ****
**F19**	H	OCH_3_	OCH_3_	OCH_3_	H	H	H	H	H	H	211.22 ± 1.23 ****
**F20**	OCH_3_	OCH_3_	H	OCH_3_	H	OCH_3_	H	OCH_3_	H	H	201.58 ± 1.14 ****
**F21**	OCH_3_	OCH_3_	H	OCH_3_	H	H	OCH_3_	OCH_3_	H	H	289.71 ± 1.86 ****
**F22**	2H	OCH_3_	H	OCH_3_	H	H	H	H	H	H	142.34 ± 1.85
**F23**	2H	OCH_3_	H	OCH_3_	H	H	OCH_3_	OCH_3_	H	H	112.01 ± 1.17
Forskolin											291.94 ± 0.82 ****
Control											100 ± 00

Significant differences compared to untreated control cells are expressed by **** *p* < 0.0001.

## Data Availability

Data are contained within the article and Supplementary Materials.

## Supplementary Materials

The following supporting information can be downloaded at https://www.mdpi.com/article/10.3390/ijms26073281/s1.
