# Supplementary material for: Methoxylated Chrysin and Quercetin as Potent Stimulators of Melanogenesis
Source: Int J Mol Sci. 2025 Apr 1;26(7):3281. doi: 10.3390/ijms26073281 (PMC11989990; doi:10.3390/ijms26073281)
Supplement: Supplementary file 1 [file ijms-26-03281-s001.zip › ijms-3538258-supplementary.pdf]

Supplementary Materials

Methoxylated Chrysin and Quercetin as Potent Stimulators of Melanogenesis

Pattara Poungcho [1,†], Rita Hairani [2,3,†], Chatchai Chaotham [4], Wanchai De-Eknamkul [1,*] and Warinthorn Chavasiri [2,*]

[1] Department of Pharmacognosy and Pharmaceutical Botany, Faculty of Pharmaceutical Sciences, Chulalongkorn University, Bangkok 10330, Thailand; 6271005433@student.chula.ac.th; wanchai.d@chula.ac.th#

[2] Center of Excellence in Natural Products Chemistry, Department of Chemistry, Faculty of Science, Chulalongkorn University, Bangkok 10330, Thailand; ritahairani@fmipa.unmul.ac.id;# warinthorn.c@chula.ac.th

[3] Department of Chemistry, Faculty of Mathematics and Natural Sciences, Mulawarman University, Samarinda 75123, East Kalimantan, Indonesia; ritahairani@fmipa.unmul.ac.id#

[4] Department of Biochemistry and Microbiology, Faculty of Pharmaceutical Sciences, Chulalongkorn University, Bangkok 10330, Thailand; chatchai.c@chula.ac.th#

* Correspondence: wanchai.d@chula.ac.th; warinthorn.c@chula.ac.th

These authors contributed equally to this work.

The effect of selected PMFs on viability of B16F10 and MNT-1cells 3
Figure S1. The effect of selected PMFs on viability of B16F10 3
Figure S2. The effect of selected PMFs on viability of MNT-1cells 4
The Elemental Analysis 5
Figure S3. ^{1}H NMR spectrum of baicalein 8
Figure S4. ^{13}C NMR spectrum of baicalein (**2**) 8
Figure S5. ^{1}H NMR spectrum of pinostrobin (**5**) 9
Figure S6. ^{13}C NMR spectrum of pinostrobin (**5**) 9
Figure S7. ^{1}H NMR spectrum of **F1** 10
Figure S8. ^{13}C NMR spectrum of **F1** 10
Figure S9. ^{1}H NMR spectrum of **F2** 11
Figure S10. ^{13}C NMR spectrum of **F2** 11
Figure S11. ^{1}H NMR spectrum of **F3** 12
Figure S12. ^{13}C NMR spectrum of **F3** 12
Figure S13. ^{1}H NMR spectrum of **F4** 13
Figure S14. ^{13}C NMR spectrum of **F4** 13
Figure S15. ^{1}H NMR spectrum of **F5** 14
Figure S16. ^{13}C NMR spectrum of **F5** 14
Figure S17. ^{1}H NMR spectrum of **F6** 15
Figure S18. ^{13}C NMR spectrum of **F6** 15
Figure S19. ^{1}H NMR spectrum of **F7** 16
Figure S20. ^{13}C NMR spectrum of **F7** 16

Figure S21. ^{1}H NMR spectrum of **F8** 17
Figure S22. ^{13}C NMR spectrum of **F8** 17
Figure S23. ^{1}H NMR spectrum of **F9** 18
Figure S24. ^{13}C NMR spectrum of **F9** 18
Figure S25. ^{1}H NMR spectrum of **F10** 19
Figure S26. ^{13}CNMR spectrum of **F10** 19
Figure S27. ^{1}H NMR spectrum of **F11** 20
Figure S28. ^{1}H NMR spectrum of **F11** 20
Figure S29. ^{1}H NMR spectrum of **F12** 21
Figure S30. ^{1}H NMR spectrum of **F13** 21
Figure S31. ^{13}C NMR spectrum of **F13** 22
Figure S32. ^{1}H NMR spectrum of **F14** 22
Figure S33. ^{1}H NMR spectrum of **F15** 23
Figure S34. ^{1}H NMR spectrum of **F16** 23
Figure S35. ^{13}C NMR spectrum of **F16** 24
Figure S36. ^{1}H NMR spectrum of **F17** 24
Figure S37. ^{1}H NMR spectrum of **F18** 25
Figure S38. ^{1}H NMR spectrum of **F19** 25
Figure S39. ^{13}C NMR spectrum of **F19** 26
Figure S40. ^{1}H NMR spectrum of **F20** 26
Figure S41. ^{1}H NMR spectrum of **F21** 27
Figure S42. ^{13}C NMR spectrum of **F21** 27
Figure S43. ^{1}H NMR spectrum of **F22** 28
Figure S44. ^{1}H NMR spectrum of **F23** 28
Figure S45. ^{13}C NMR spectrum of **F23** 29

The effect of selected PMFs on viability of B16F10 and MNT-1cells

The effect of some selected PMFs with potent melanogenetic activity (**F1**, **F16**, and **F19-F21)** on the viability of B16F10 and MNT-1 cells was assessed. This was carried out by MTT assay method in which cells were treated with various concentrations of the compounds for 48 h The results showed that all the selected potent compounds t showed no cytotoxicity at any concentrations from 1 to 10 μM on B16F10 (of **F1**, **F16**, and **F19-F21**) and MNT-1 (of **F1**, **F16, F19** and **F21**) cells (Figure S-1 and Figure S-2).

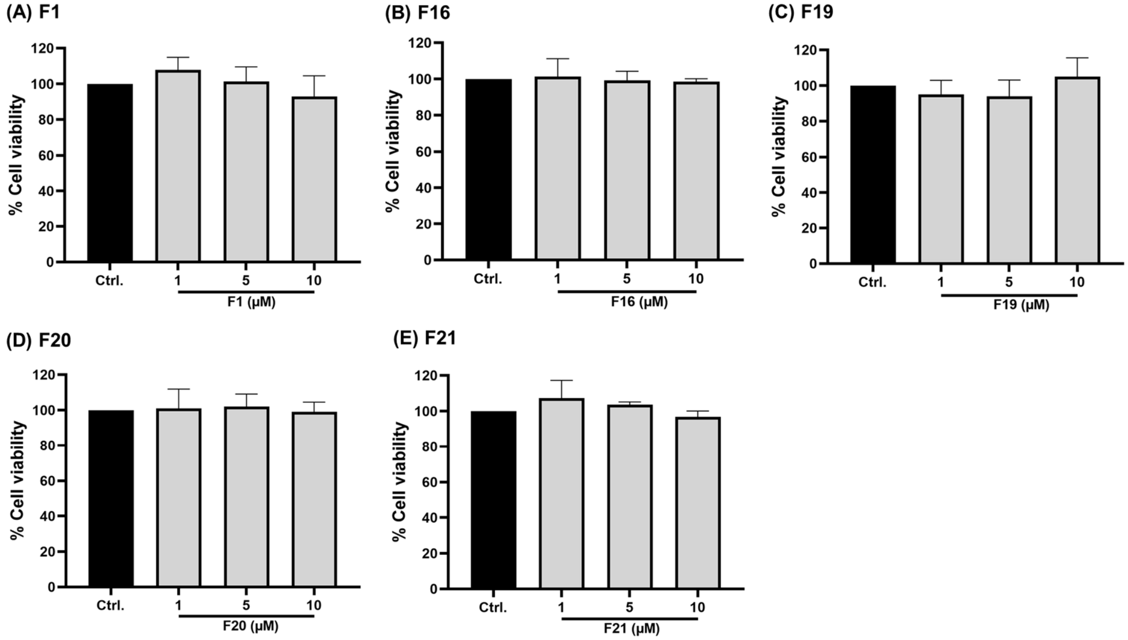

Figure S1. Effect of some synthesized PMFs with potent melanogenetic activity (**F1**, **F16**, and **F19-F21**) on the cytotoxicity of B16F10 cells. Cell viability was measured by the MTT assay method after 24 h of incubation with the compounds: (A) **F1**; (B) **F16**; (C) **F19**; (D) **F20** and (E) **F21**. Data are expressed as a percentage of the number of viable cells observed in the control group, and each bar graph presents the mean value ± SD from three independent experiments.

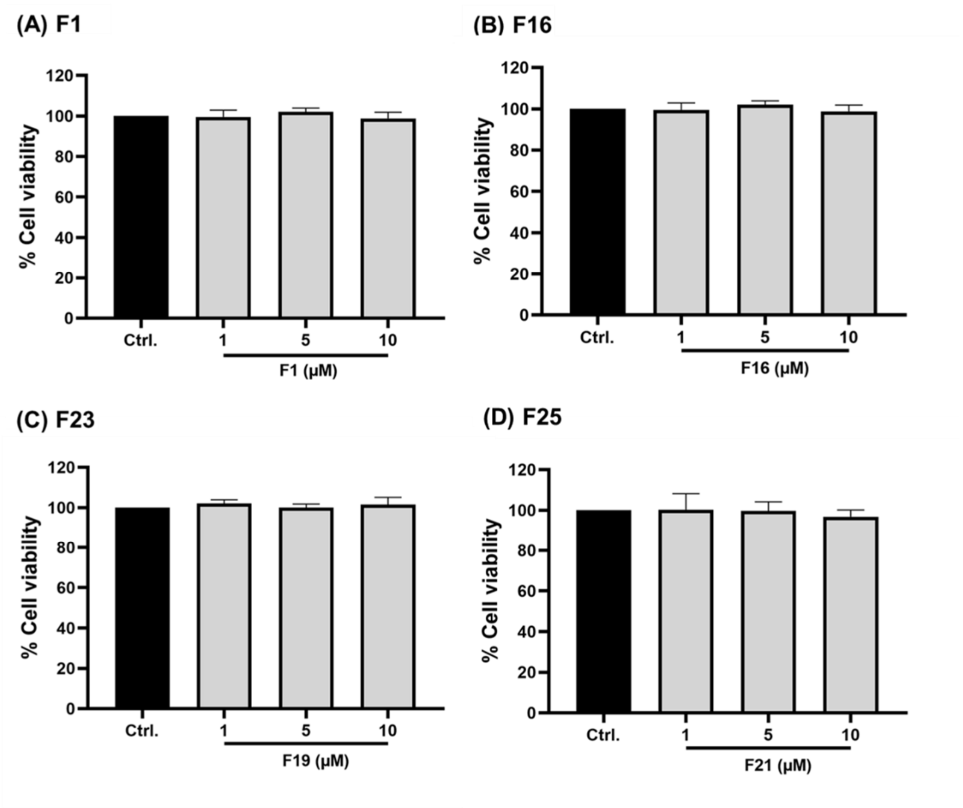

Figure S2. Effect of **F1** and **F21** on the cytotoxicity of MNT-1 cells. Cell viability was measured by the MTT assay method after 24 h of incubation: (A) **F1**; (B) **F16**; (C) **F19**; and (D) **F21**. Data are expressed as a percentage of the number of viable cells observed in the control group, and each column presents the mean values ± SD from three independent experiments performed in triplicate.

The Elemental Analysis

Baicalein (**F2**): yellow solid (0.1%), ^{1}H NMR (500 MHz, DMSO-*d*$_6$) δ (ppm) 8.06 (dd, J = 8.0, 1.5 Hz, 2H), 7.58 (m, 3H), 6.93 (s, 1H), and 6.63 (s, 1H). ^{13}C NMR (125 MHz, DMSO-*d*$_6$) δ (ppm) 182.2, 163.0, 153.7, 149.9, 147.0, 131.9, 131.0, 129.4, 129.2, 126.4, 104.5, 104.3, and 94.1.

Pinocembrin (**F5**): pale-yellow solid (16%), ^{1}H NMR (500 MHz, Acetone-*d*$_6$) δ (ppm) 12.16 (s, 5-OH), 9.76 (s, 7-OH), 7.57 (dd, *J* = 7.0, 1.5 Hz, 2H), 7.42 (m, 3H), 6.00 (d, *J* = 2.5 Hz, 1H), 5.97 (d, *J* = 2.0 Hz, 1H), 5.57 (dd, *J* = 13.0, 3.5 Hz, 1H), 3.17 (dd, *J* = 17.0, 12.5 Hz, 1H), and 2.81 (dd, *J* = 17.0, 3.0 Hz, 1H); ^{13}C NMR (125 MHz, Acetone-*d6*) δ (ppm) 196.8, 167.4, 165.2, 164.1, 140.0, 129.4, 127.3, 103.2, 96.9, 95.9, 79.9, and 43.5.

5,7-Dimethoxyflavone (**F1**): white powder (40%). ^{1}H NMR ($CDCl_3$) δ (ppm) 7.87 (dd, *J* = 7.0, 2.0 Hz, 2H), 7.49 (m, 3H), 6.68 (s, 1H), 6.57 (d, *J* = 2.0 Hz, 1H), 6.37 (d, *J* = 3.5 Hz, 1H), 3.95 (s, 3H), and 3.91 (s, 3H). ^{13}C NMR ($CDCl_3$) δ (ppm) 177.8, 164.2, 161.0, 160.9, 160.1, 131.6, 131.4, 129.1, 126.1, 109.4, 109.1, 96.3, 93.0, 56.6, and 55.9.

5,7-Diethoxyflavone (**F2**): white powder (40%). ^{1}H NMR (acetone-*d*$_6$) δ (ppm) 8.00 (dd, *J* = 6.5, 2.5 Hz, 2H), 7.57 (m, 3H), 6.74 (s, 1H), 6.60 (d, *J* = 2.0 Hz, 1H), 6.38 (d, *J* = 2.3 Hz, 1H), 6.44 (s, 1H), 4.19 (m, 2H), 4.12 (m, 2H), and 1.42 (m, 6H). ^{13}C NMR (acetone-*d*$_6$) δ (ppm) 176.4, 164.2, 161.0, 160.7, 160.6, 132.5, 131.9, 129.8, 126.7, 109.9, 109.3, 98.1, 94.3, 65.4, 64.8, and 14.8.

7-Methoxy-5-hydroxyflavone (**F3**): yellow powder (53%). ^{1}H NMR ($CDCl_3$) δ (ppm) 7.87 (dd, *J* = 8.0, 1.5 Hz, 2H), 7.52 (m, 3H), 6.65 (s, 1H), 6.48 (d, *J* = 2.0 Hz, 1H), 6.36 (d, *J* = 2.0 Hz, 1H), and 3.87 (s, 3H). ^{13}C NMR ($CDCl_3$) δ (ppm) 182.6, 165.7, 164.1, 162.3, 157.9, 132.0, 131.5, 129.2, 126.4, 105.9, 105.8, 98.3, 92.8, and 55.9.

7-Ethoxy-5-hydroxyflavone (**F4**): yellow powder (69%). ^{1}H NMR ($CDCl_3$) δ (ppm) 7.88 (dd, *J* = 8.0, 1.5 Hz, 2H), 7.52 (m, 3H), 6.66 (s, 1H), 6.48 (d, *J* = 2.5 Hz, 1H), 6.36 (d, *J* = 2.0 Hz, 1H), 4.11 (q, *J* = 7.0 Hz, 2H), and 1.45 (t, *J* = 7.0 Hz, 3H). ^{13}C NMR ($CDCl_3$) δ (ppm) 182.6, 165.2, 164.1, 162.3, 157.9, 132.0, 131.5, 129.2, 126.4, 105.9, 105.7, 98.7, 93.2, 64.4, and 14.7.

7-Butoxy-5-hydroxyflavone (**F5**): pale yellow powder (58%). ^{1}H NMR ($CDCl_3$) δ (ppm) 7.88 (dd, *J* = 8.0, 1.5 Hz, 2H), 7.53 (m, 3H), 6.66 (s, 1H), 6.49 (d, *J* = 2.0 Hz, 1H), 6.36 (d, *J* = 2.0 Hz, 1H), 4.04 (t, *J* = 7.0 Hz, 2H), 1.80 (m, 2H), 1.51 (m, 2H), and 0.99 (t, *J* = 7.0 Hz, 3H). ^{13}C NMR ($CDCl_3$) δ (ppm) 182.6, 165.4, 164.1, 162.2, 157.9, 132.0, 129.2, 126.4, 106.0, 105.7, 98.8, 93.2, 68.5, 31.1, 19.3, and 13.9.

7-Hexyloxy-5-hydroxyflavone (**F6**): pale yellow powder (76%). ^{1}H NMR ($CDCl_3$) δ (ppm) 7.87 (dd, *J* = 8.0, 1.5 Hz, 2H), 7.52 (m, 3H), 6.65 (s, 1H), 6.48 (d, *J* = 2.5 Hz, 1H), 6.35 (d, *J* = 2.0 Hz, 1H), 4.02 (t, *J* = 6.5 Hz, 2H), 1.81 (m, 2H), 1.46 (m, 2H), 1.35 (m, 4H), and 0.92 (t, *J* = 7.0 Hz, 3H). ^{13}C NMR ($CDCl_3$) δ (ppm) 182.6, 165.4, 164.0, 162.2, 157.9, 131.9, 131.5, 129.2, 126.4, 105.9, 105.7, 98.7, 93.2, 68.8, 31.6, 29.0, 25.7, 22.7, and 14.2.

5-Hydroxy-7-octyloxyflavone (**F7**): pale yellow powder (51%). ^{1}H NMR ($CDCl_3$) δ (ppm) 7.87 (dd, *J* = 8.0, 1.5 Hz, 2H), 7.53 (m, 3H), 6.66 (s, 1H), 6.49 (d, *J* = 2.0 Hz, 1H), 6.36 (d, *J* = 2.5 Hz, 1H), 4.02 (t, *J* = 6.5 Hz, 2H), 1.81 (m, 2H), 1.46 (m, 2H), 1.32 (m, 8H), and 0.89 (t, *J* = 7.0 Hz, 3H). ^{13}C NMR ($CDCl_3$) δ (ppm) 182.6, 165.3, 164.0, 162.2, 157.9, 131.9, 131.5, 129.2, 126.4, 106.0, 105.7, 98.8, 93.2, 68.9, 31.9, 29.4, 29.3, 29.1, 26.1, 22.8, and 14.2.

7-Ethoxy-5-methoxyflavone (**F8**): yellow powder (69%). ^{1}H NMR (DMSO-*d6*) δ (ppm) 8.02 (dd, *J* = 7.5, 1.5 Hz, 2H), 7.55 (m, 3H), 6.82 (d, *J* = 2.0 Hz, 1H), 6.75 (s, 1H), 6.47 (d, *J* = 2.5 Hz, 1H), 4.16 (q, *J* = 7.5 Hz, 2H), 3.82 (s, 3H), and 1.37 (t, *J* = 7.0 Hz, 3H). ^{13}C NMR (DMSO-*d6*) δ (ppm) 175.7, 163.1, 160.3, 159.6, 159.2, 131.4, 130.9, 129.1, 125.9, 108.3, 108.2, 96.6, 93.7, 64.1, 56.1, and 14.4.

7-Butoxy-5-methoxyflavone (**F9**): pale yellow powder (58%). ^{1}H NMR (DMSO-*d6*) δ (ppm) 8.03 (dd, *J* = 7.0, 1.5 Hz, 2H), 7.55 (m, 3H), 6.85 (d, *J* = 3.0 Hz, 1H), 6.76 (s, 1H), 6.47 (d, *J* = 2.0 Hz, 1H), 4.10 (t, *J* = 6.5 Hz, 2H), 3.82 (s, 3H), 1.73 (m, 2H), 1.45 (m, 2H), and 0.95 (t, *J* = 7.5 Hz, 3H). ^{13}C NMR (DMSO-*d6*) δ (ppm) 175.7, 163.2, 160.3, 159.5, 159.2, 131.4, 130.9, 129.1, 125.9, 108.3, 108.2, 96.6, 93.7, 68.1, 56.1, 30.5, 18.7, and 13.7.

7-Hexyloxy-5-methoxyflavone (**F10**): pale yellow powder (76%). ^{1}H NMR ($CDCl_3$) δ (ppm) 7.86 (dd, *J* = 7.0, 2.0 Hz, 2H), 7.49 (m, 3H), 6.68 (s, 1H), 6.55 (d, *J* = 2.0 Hz, 1H), 6.37 (d, *J* = 2.5 Hz, 1H), 4.05 (t, *J* = 6.5 Hz, 2H), 3.95 (s, 3H), 1.82 (m, 2H), 1.49 (m, 2H), 1.36 (m, 4H), and 0.92 (t, *J* = 7.5 Hz, 3H). ^{13}C NMR ($CDCl_3$) δ (ppm) 177.8, 163.8, 161.0, 160.8, 160.0, 131.7, 131.3, 129.1, 126.1, 109.2, 109.1, 96.7, 93.4, 68.8, 56.5, 31.6, 29.1, 25.8, 22.7, and 14.1

5-Methoxy-7-octyloxyflavone (**F11**): pale yellow powder (51%). ^{1}H NMR ($CDCl_3$) δ (ppm) 7.86 (dd, *J* = 7.5, 2.05 Hz, 2H), 7.49 (m, 3H), 6.55 (d, *J* = 2.0 Hz, 1H), 6.37 (d, *J* = 2.5 Hz, 1H), 4.05 (t, *J* = 6.5 Hz, 2H), 3.95 (s, 3H), 1.82 (m, 2H), 1.48 (m, 2H), 1.33 (m, 8H), and 0.89 (t, *J* = 6.5 Hz, 3H). ^{13}C NMR ($CDCl_3$) δ (ppm) 177.8, 163.8, 161.0, 160.1, 131.7, 131.3, 129.1, 126.1, 109.2, 109.1, 96.7, 93.4, 68.8, 56.6, 31.9, 29.4, 29.3, 29.1, 26.1, 22.8, and 14.2.

2′,5,7-Trimethoxyflavone (**F12**): pale yellow solid (88%). ^{1}H NMR ($CDCl_3$) δ (ppm) 7.87 (d, *J* = 7.9 Hz, 2H), 7.45 (t, *J*= 7.6 Hz, 2H), 7.02 (s, 1H), 6.54 (d, *J* = 2.3 Hz, 1H), 6.36 (d, *J* = 2.3 Hz, 1H), 4.01-3.85 (m, 9H).

4′,5,7-Trimethoxyflavone (**F13**): white powder (83%). ^{1}H NMR (acetone-*d6*) δ (ppm) 8.00 (dd, *J* = 6.5, 2.5 Hz, 2H), 7.57 (m, 3H), 6.74 (s, 1H), 6.60 (d, *J* = 2.0 Hz, 1H), 6.38 (d, *J* = 2.3 Hz, 1H), 6.44 (s, 1H), 4.19 (m, 2H), 4.12 (m, 2H), and 1.42 (m, 6H). ^{13}C NMR (acetone-*d6*) δ (ppm) 176.4, 164.2, 161.0, 160.7, 160.6, 132.5, 131.9, 129.8, 126.7, 109.9, 109.3, 98.1, 94.3, 65.4, 64.8, and 14.8.

2′,4′,5,7-Tetramethoxyflavone (**F14**): pale brown solid (92%). ^{1}H NMR ($CDCl_3$) δ (ppm) 7.85 (d, *J* = 8.7 Hz, 1H), 6.61 (dd, *J*= 8.8, 2.4 Hz, 2H), 6.53 (d, *J* = 2.6 Hz, 2H), 6.36 (d, *J* = 2.3 Hz, 1H), 3.91 (dd, *J* = 19.0, 8.5 Hz, 12H).

2′,5,5′,7-Tetramethoxyflavone (**F15**): pale brown solid (87%). ^{1}H NMR ($CDCl_3$) δ (ppm) 7.44 (d, *J* = 3.2 Hz, 2H), 6.99 (m, 2H), 6.57 (d, *J* = 2.3 Hz, 1H), 6.38 (d, *J* = 2.3 Hz, 1H), 4.19 – 3.75 (m, 12H).

3′,4′,5,7-Tetramethoxyflavone (**F16**): pale yellow powder (80%). ^{1}H NMR ($CDCl_3$) δ (ppm) 7.64 (dd, J = 2.5, 2.5 Hz, 1H), 7.52 (d, J = 2 Hz, 1H), 6.87 (d, J = 2.5 Hz, 1H), 6.77 (s, 1H), 6.50 (d, J = 2.0 Hz, 1H), 3.90 (s, 3H), 3.88 (s, 3H), 3.84 (s, 3H), and 3.82 (s, 3H). ^{13}C NMR ($CDCl_3$) δ (ppm) 175.9, 163.7, 160.3, 159.8, 159.3, 151.6, 149.1, 123.2, 119.4, 111.7, 109.1, 108.3, 107.1, 96.3, 93.5, 56.1, 56.1, 55.9, and 55.8.

2′,3′,4′,5,7-Pentamethoxyflavone (**F17**): pale brown solid (91%). ^{1}H NMR ($CDCl_3$) δ (ppm) 7.48 (d, *J* = 8.9 Hz, 1H), 6.77 (s, 1H), 6.71 (d, *J* = 8.9 Hz, 1H), 6.49 (d, *J* = 2.4 Hz, 1H), 6.35 (d, *J* = 15.5 Hz, 1H), 4.12 – 3.70 (m, 15H).

3′,4′,5,5′,7-Pentamethoxyflavone (**F18**): brown solid (86%). ^{1}H NMR ($CDCl_3$) δ (ppm) 7.10 (s, 2H), 6.90 (s, 1H), 6.61 (d, *J* = 2.3 Hz, 1H), 6.40 (d, *J* = 2.2 Hz, 1H), 4.17 – 3.77 (m, 15H).

5,6,7-Trimethoxyflavone (**F19**): white powder (78%). ^{1}H NMR ($CDCl_3$) δ (ppm) 7.89 (m, 2H), 7.51 (m, 3H), 6.83 (s, 1H), 6.76 (s, 1H), 3.99 (s, 6H), and 3.92 (s, 3H). ^{13}C NMR ($CDCl_3$) δ (ppm) 177.5, 161.6, 158.1, 154.8, 152.7, 140.6, 131.6, 129.1, 126.2, 112.9, 108.3, 96.4, 62.4, 61.7, and 56.5.

2′,3,4′,5,7-Pentamethoxyflavone (**F20**): white powder (53%). ^{1}H NMR (DMSO-*d6*) δ (ppm) 7.64 (m, 2H), 7.12 (d, J = 10 Hz), 6.80 (s, 1H),6.47 (s, 1H), 3.88 (s, 3H), 3.85 (s, 3H), 3.84 (s, 3H), 3.83 (s, 3H), and 3.74 (s, 3H).

3,3′,4′,5,7-Pentamethoxyflavone (**F21**): white powder (69%). ^{1}H NMR ($CDCl_3$) δ (ppm) 7.70 (m, 2H), 6.97 (d, J = 7.5 Hz, 1H), 6.50 (d, J = 2.5 Hz, 1H),6.34 (d, J = 2.0 Hz, 1H), 3.96 (s, 9H), 3.90 (s, 3H), and 3.87 (s, 3H). ^{13}C NMR ($CDCl_3$) δ (ppm) 174.2, 164.0, 161.1, 158.9, 152.7, 150.9, 148.8, 141.3, 123.5, 121.8, 111.4, 110.9, 109.6, 95.9, 92.6, 60.1, 56.5, 56.2, 56.1, and 55.9.

5,7-Dimethoxyflavanone (**F22**): white powder (58%). ^{1}H NMR ($CDCl_3$) δ (ppm) 7.89 (d, J = 20 Hz, 1H), 7.78 (d, J = 15 Hz, 1H), 7.62 (d, J = 5 Hz, 2H), 7.42 (m, 5H), 6.01 (s, 1H), 3.96 (s, 3H), and 3.91 (s, 3H).

3′,4′,5,7-Tetramethoxyflavanone (**F23**): white powder (76%). ^{1}H NMR ($CDCl_3$) δ (ppm) 6.99 (d, J = 10 Hz, 2H), 6.89 (m, 1H), 6.15 (d, J = 2.3 Hz, 1H), 6.09 (d, J = 2.3 Hz, 1H), 5.34 (dd, J = 2.9, 2.6 Hz, 1H), 3.92 (s, 3H), 3.89 (s, 6H), 3.82 (s, 3H), 3.04 (dd, J = 13.45, 13.15 Hz, 1H), and 2.77 (dd, J = 2.9, 2.9 Hz, 1H). ^{13}C NMR ($CDCl_3$) δ (ppm) 189.6, 166.1, 165.1, 162.4, 149.5, 149.3, 131.3, 119.0, 111.2, 109.4, 106.1, 93.7, 93.3, 79.3, 56.3, 56.1, 55.7, and 55.6.

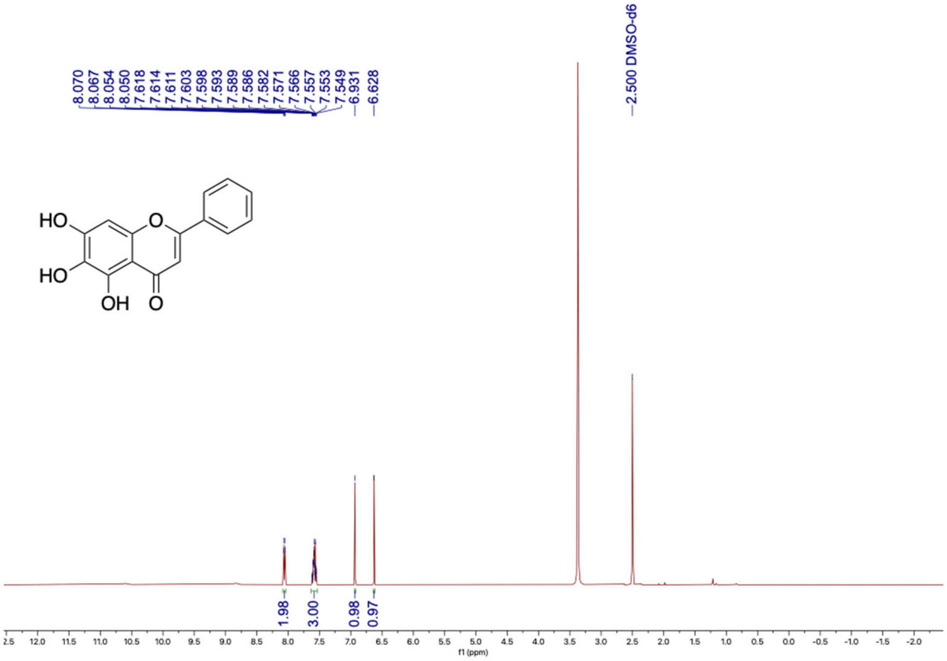

Ijxuh#V61#K#QPU#vshfwuxp#ri#edlfdohlq#5,#

#

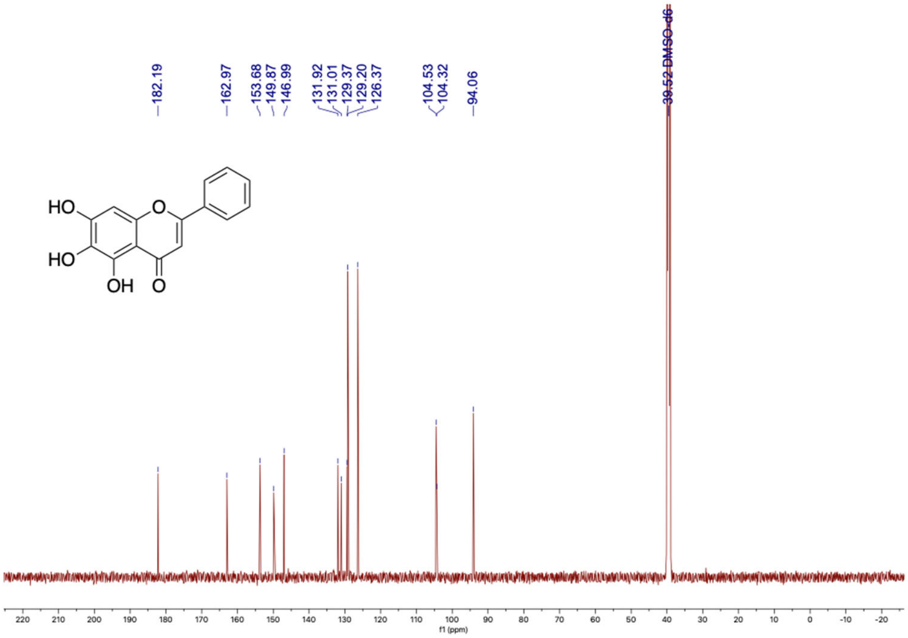

Ijxuh#V71#46F#QPU#vshfwuxp#ri#edlfdohlq#5,

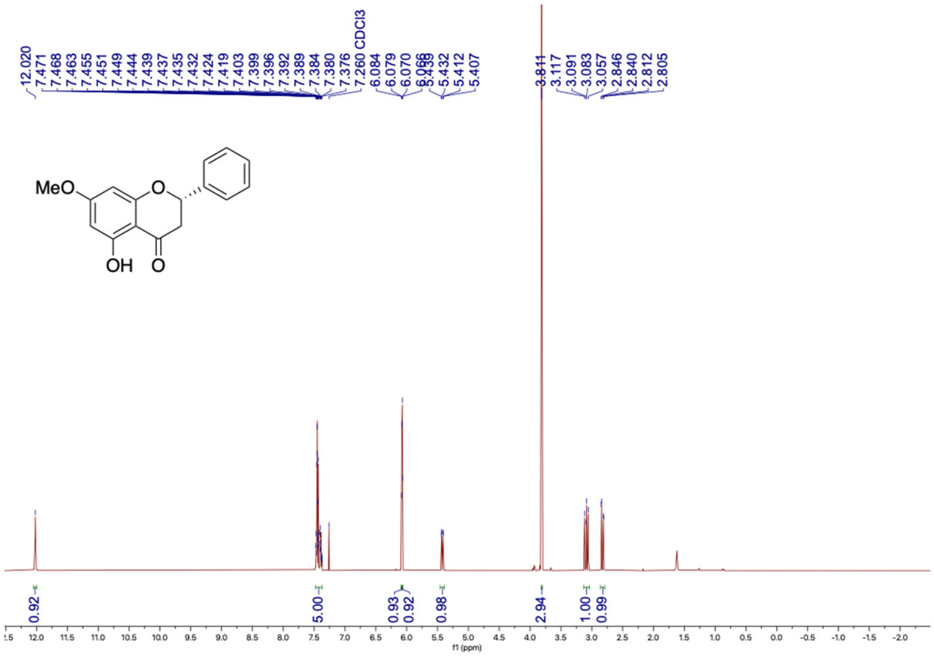

Ljxuh#V81#4K#QPU#vshfwuxp#ri#slqrvwurelq#+8,#

#

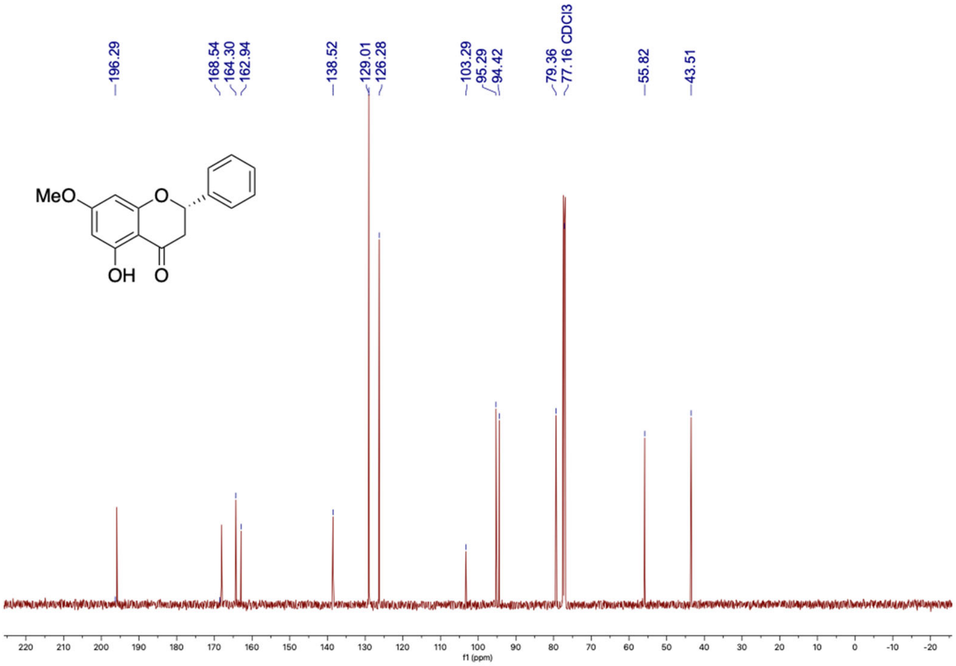

Ljxuh#V91#46F#QPU#vshfwuxp#ri#slqrvwurelq#+8,#

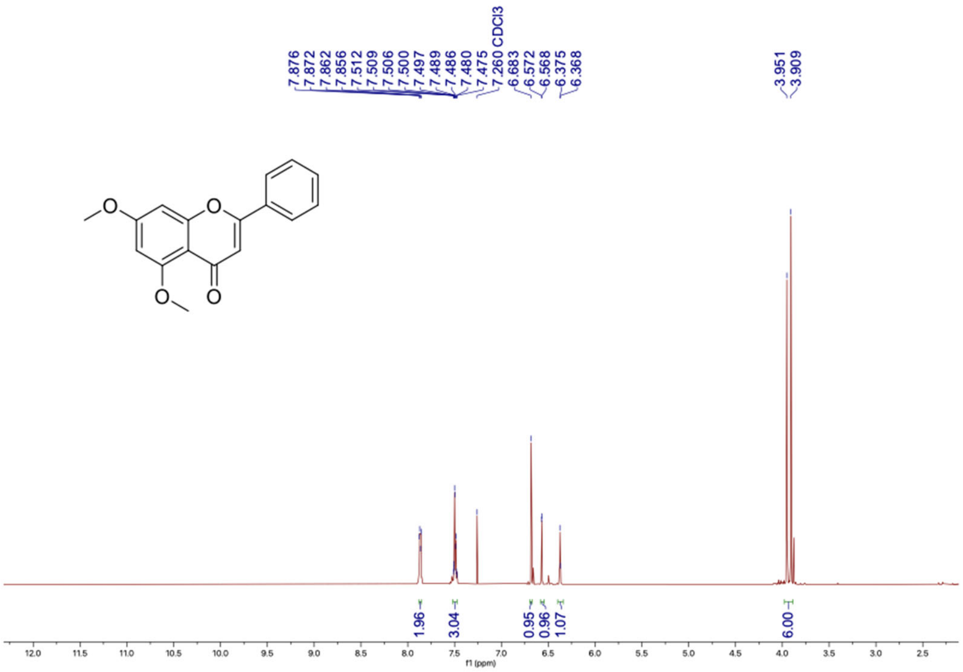

Ljxuh#V:1#4K#QPU#vshfwuxp#ri#I4#

#

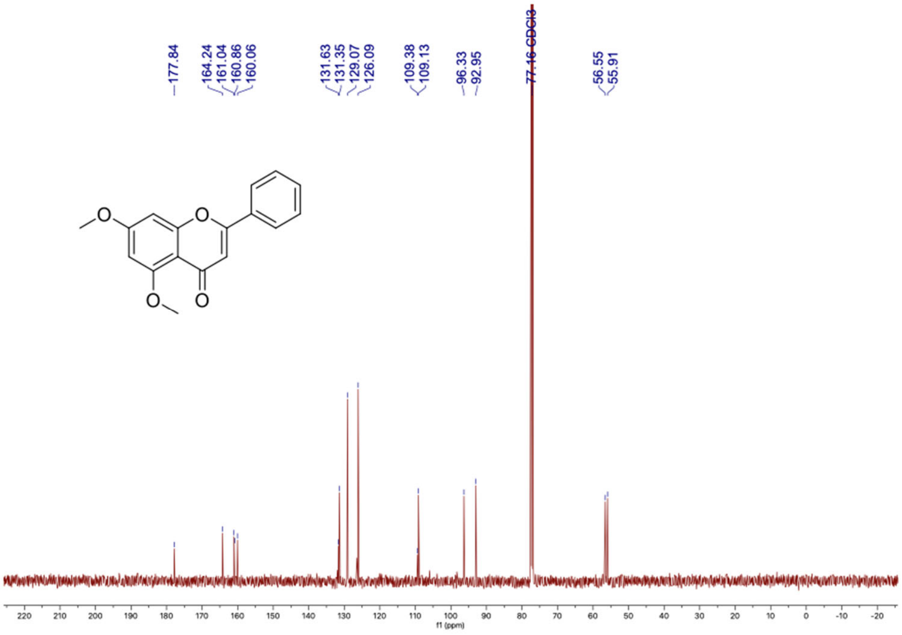

Ljxuh#V;1#46F#QPU#vshfwuxp#ri#I4#

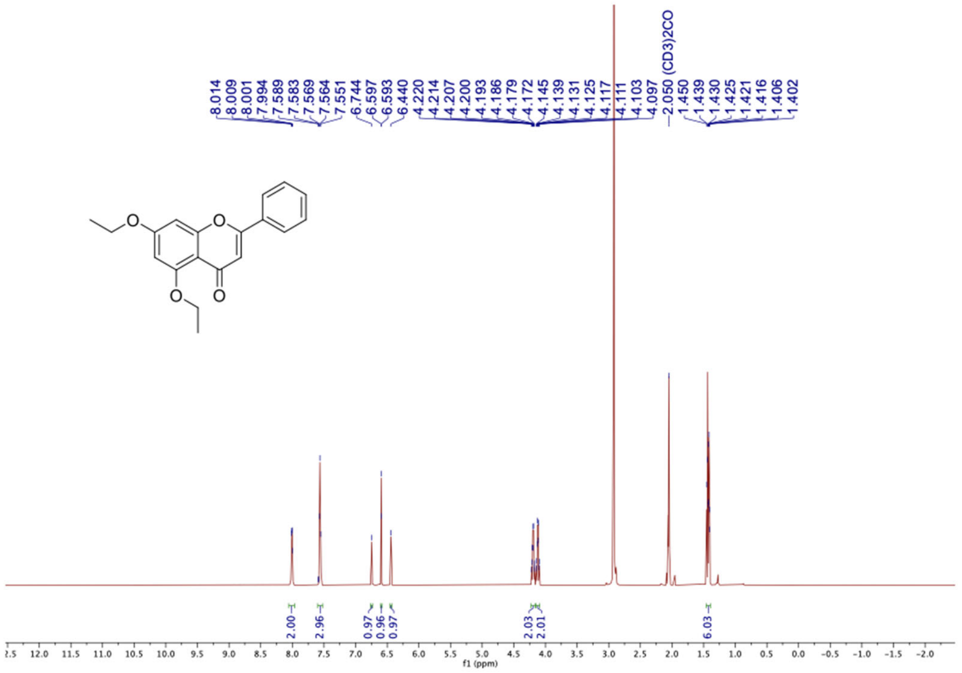

#

Iljxuh#V<1#4K#QPU#vshfwuxp#ri#I5#

#

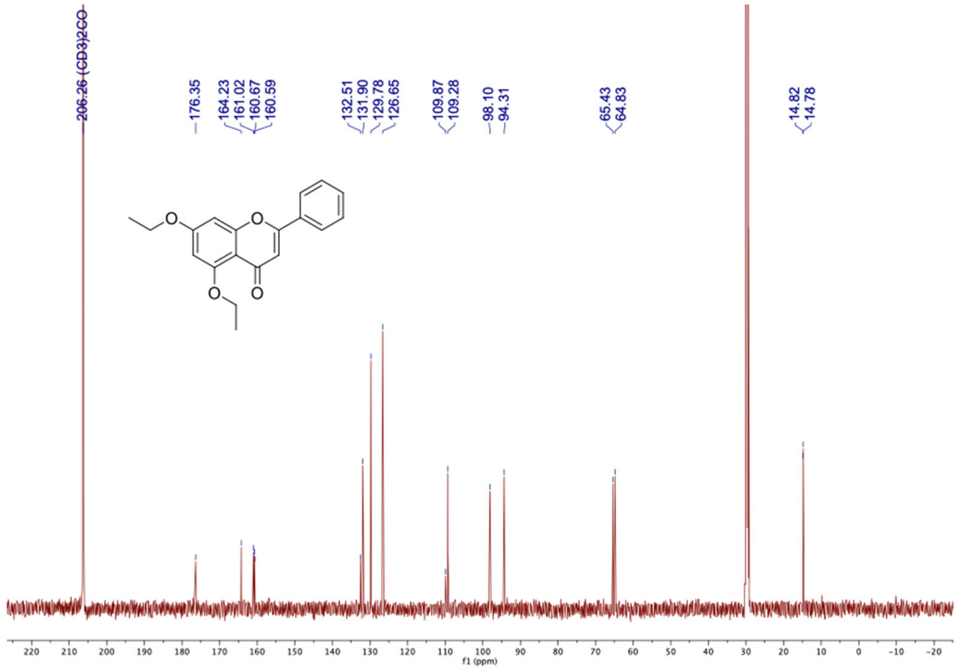

#

Iljxuh#V431#46F#QPU#vshfwuxp#ri#I5#

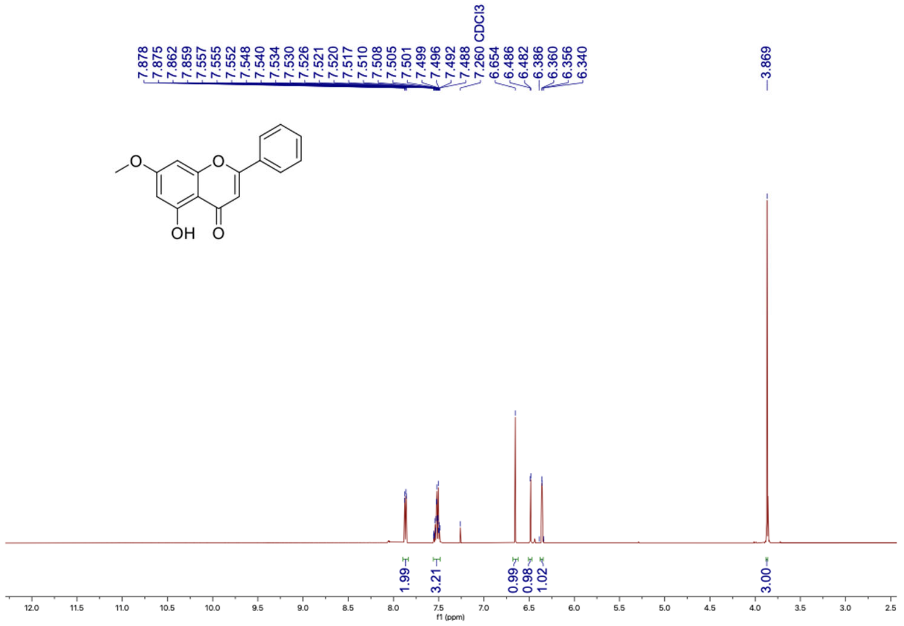

#

Iljxuh#V441#^{4}K#QPU#vshfwuxp#ri#I6#

#

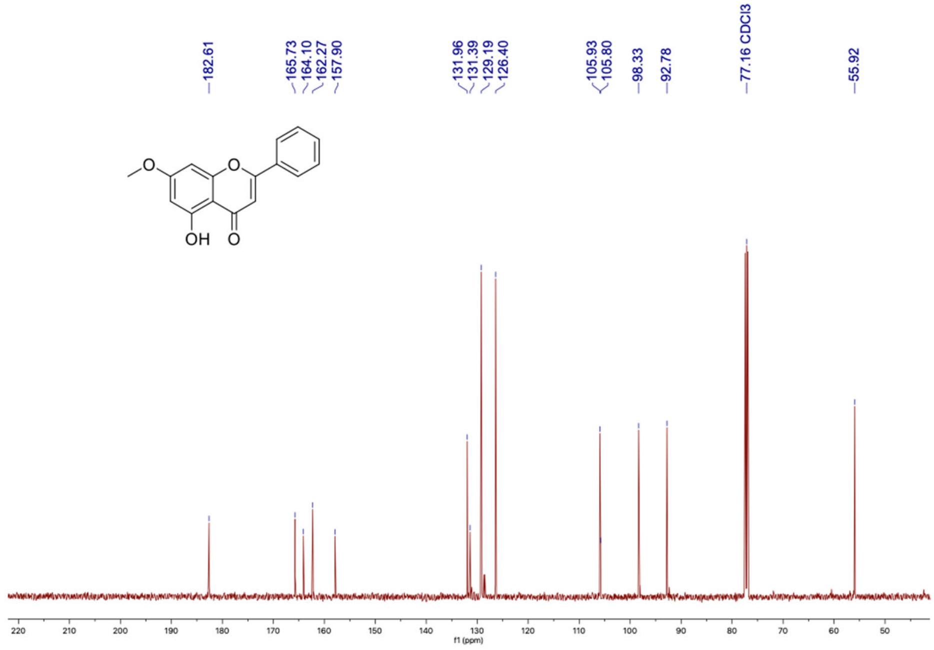

#

Iljxuh#V451#^{46}F#QPU#vshfwuxp#ri#I6#

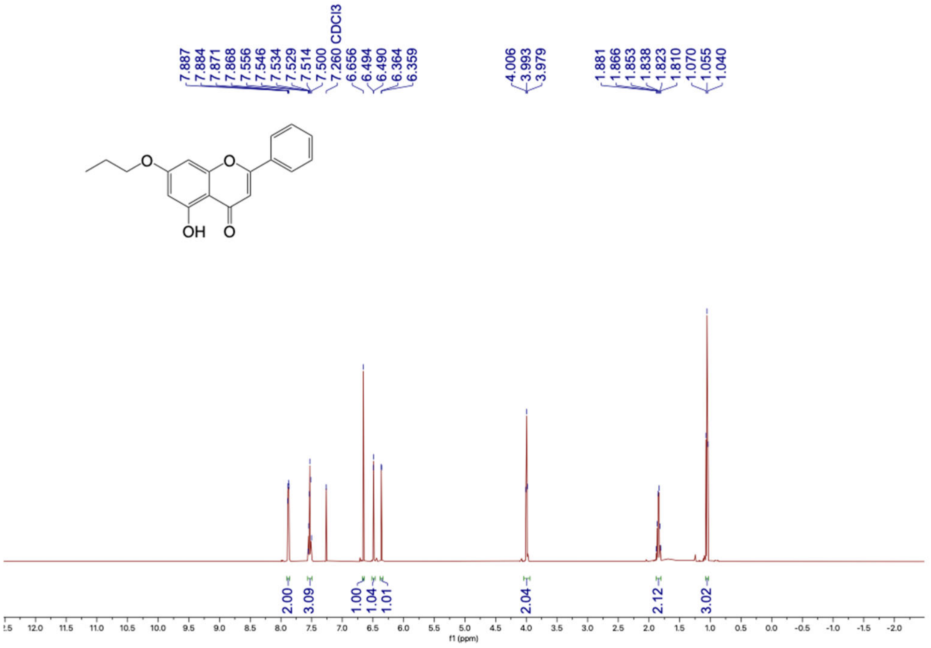

#

Ijxuh#V461#^{4}K#QPU#vshfwuxp#ri#I7#

#

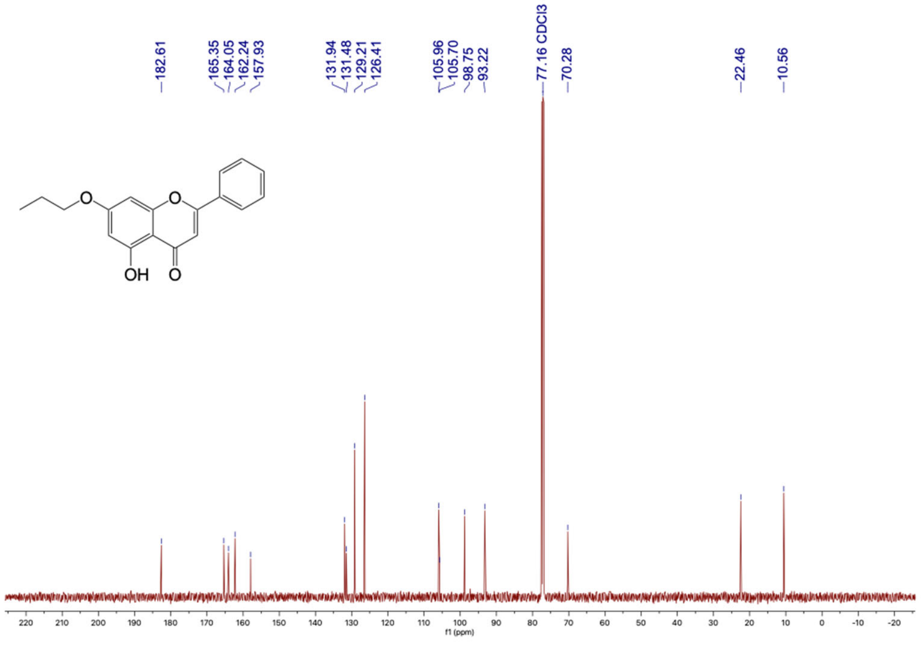

#

Ijxuh#V471#^{46}F#QPU#vshfwuxp#ri#I7#

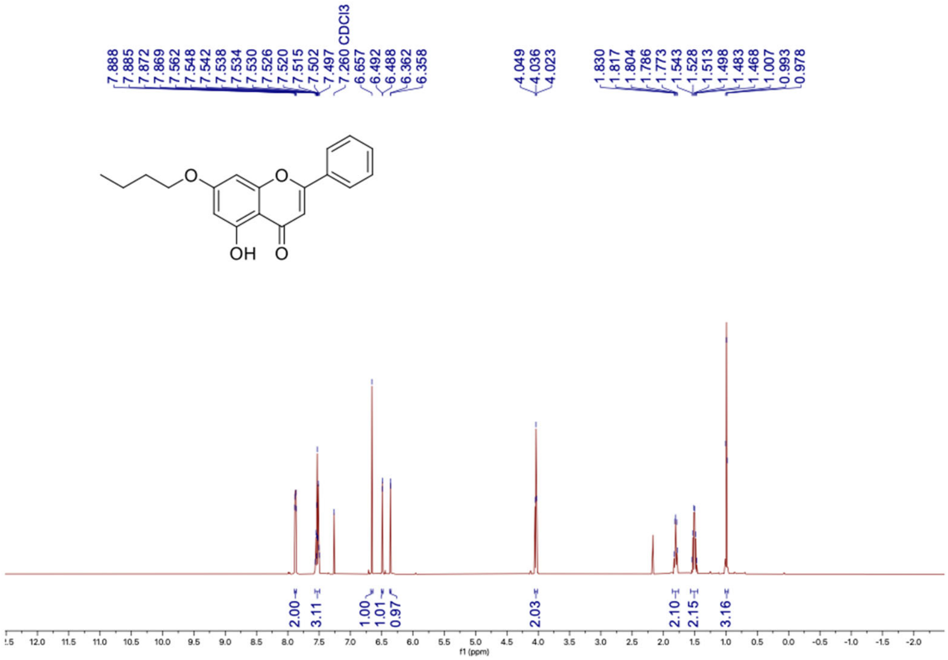

#

Ljxuh#V481#4K#QPU#vshfwuxp#ri#I8#

#

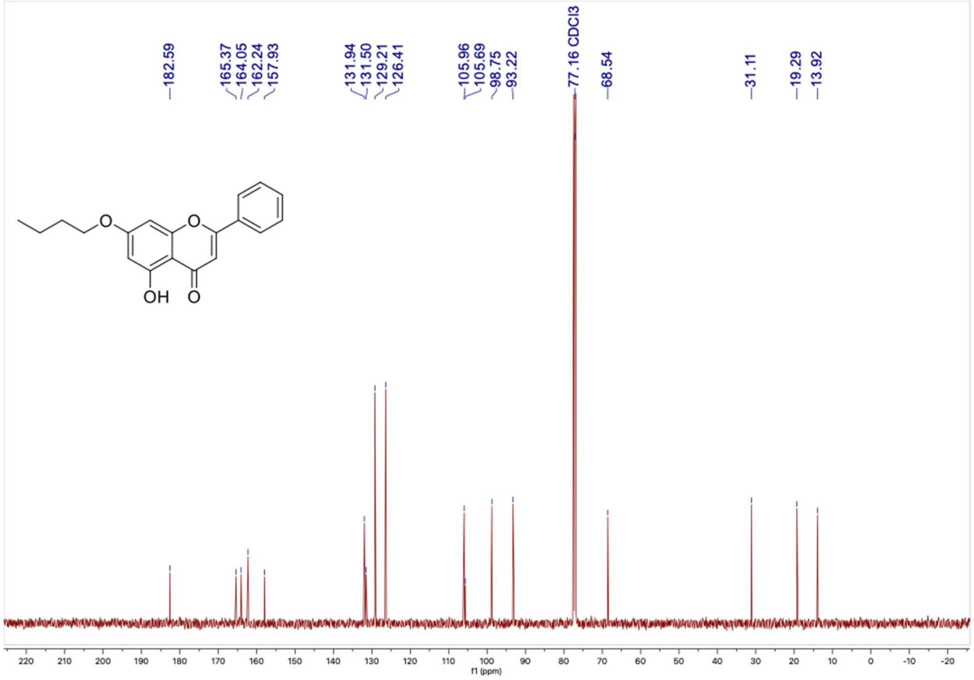

#

Ljxuh#V491#46F#QPU#vshfwuxp#ri#I8#

#

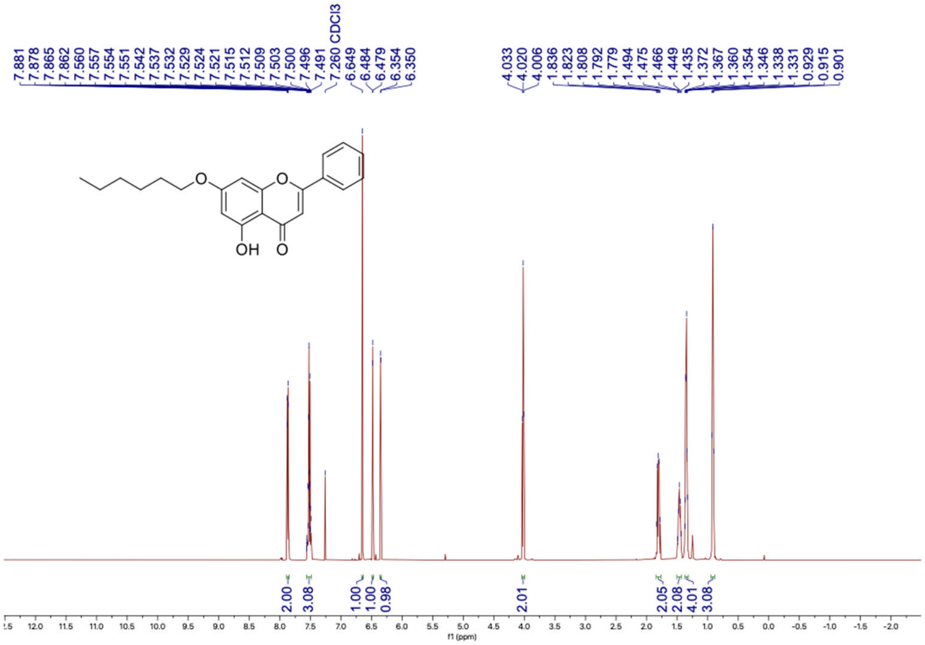

#

Ljxuh#V4:1#[4]K#QPU#vshfwuxp#ri#I9#

#

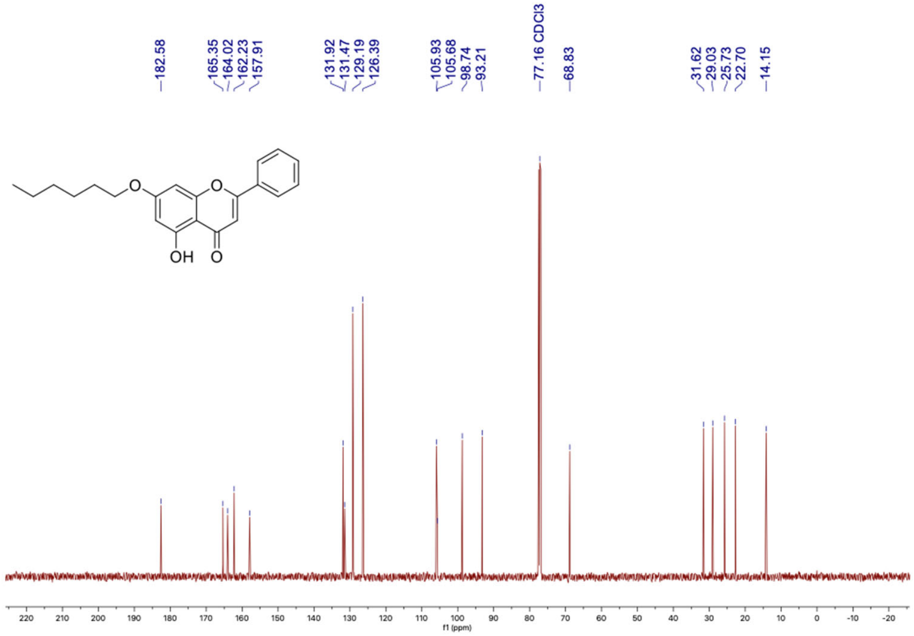

#

Ljxuh#V4;1#[46]F#QPU#vshfwuxp#ri#I9#

#

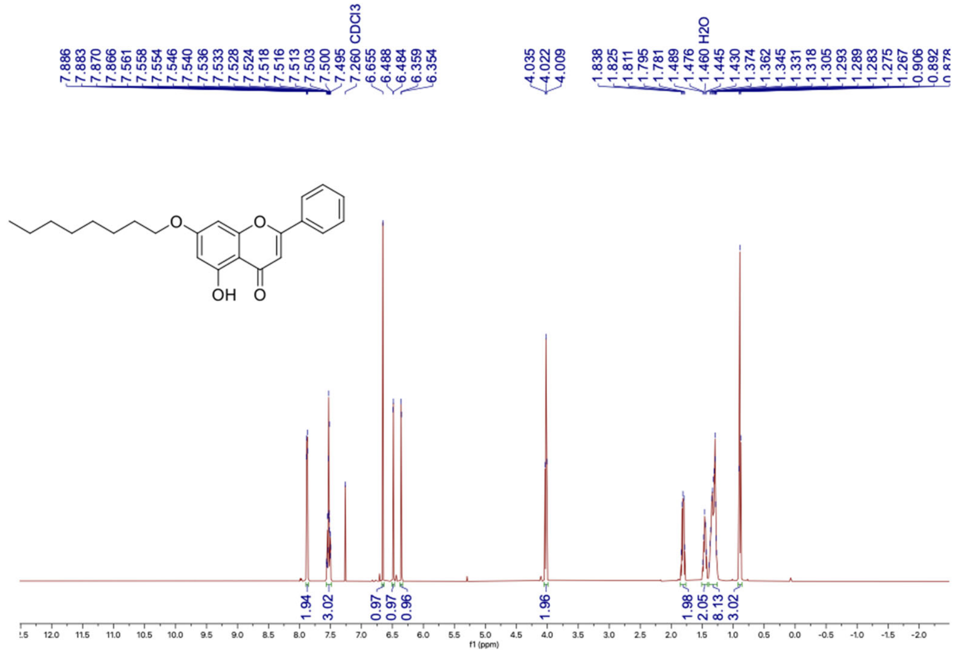

#

Ljxuh#V4<1#4K#QPU#vshfwuxp#ri#I:#

#

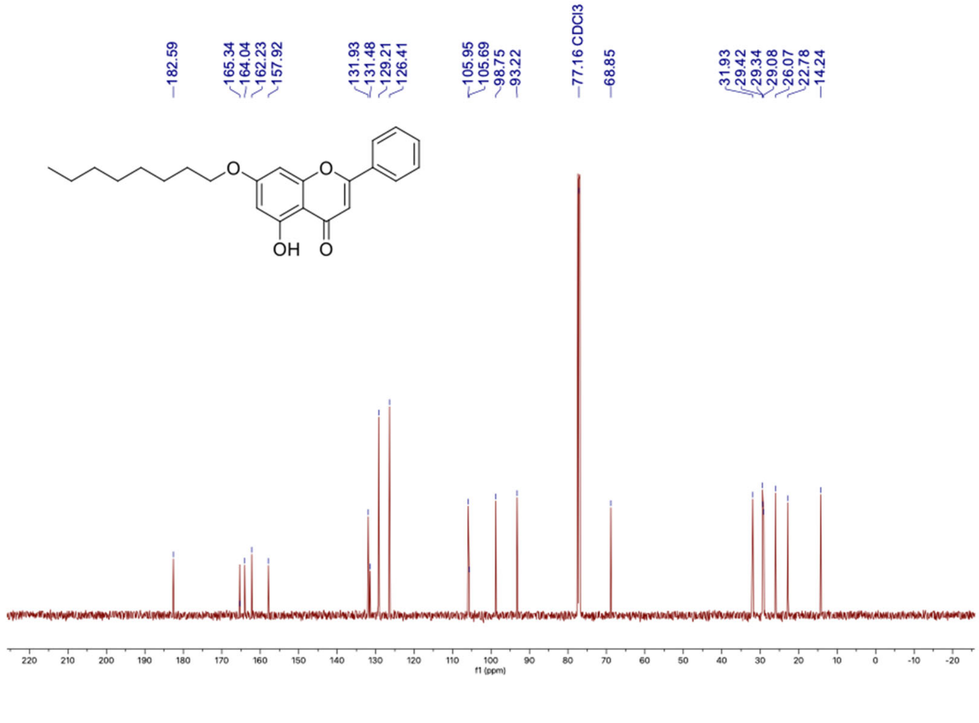

#

Ljxuh#V531#46F#QPU#vshfwuxp#ri#I:#

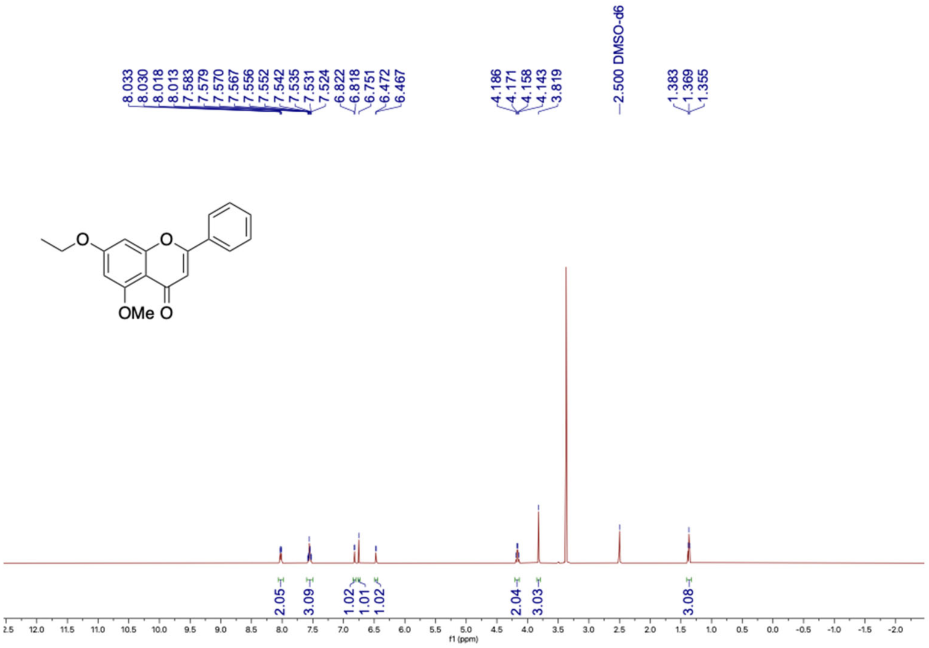

#

Iljxuh#V54#4K#QPU#vshfwuxp#ri#I;#

#

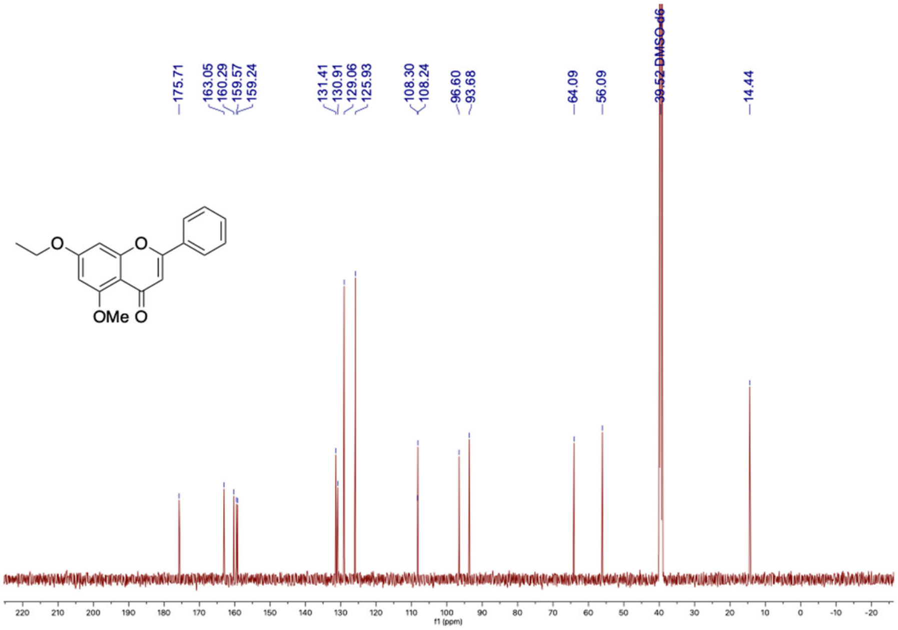

#

Iljxuh#V55#46F#QPU#vshfwuxp#ri#I;#

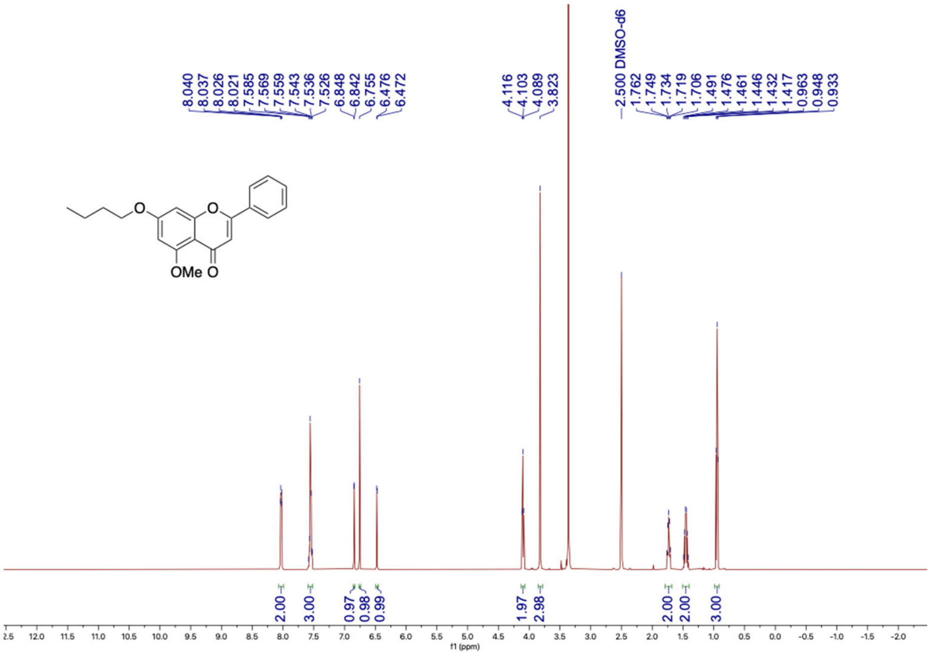

#

Ijxuh#V561#K#QPU#vshfwuxp#ri#I<#

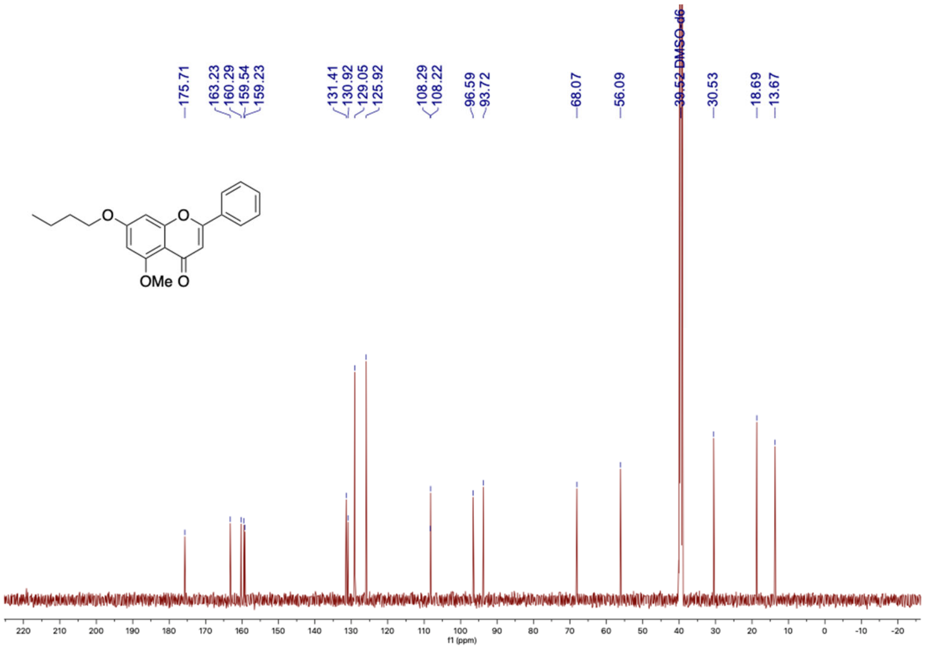

#

Ijxuh#V571#K#QPU#vshfwuxp#ri#I<#

#

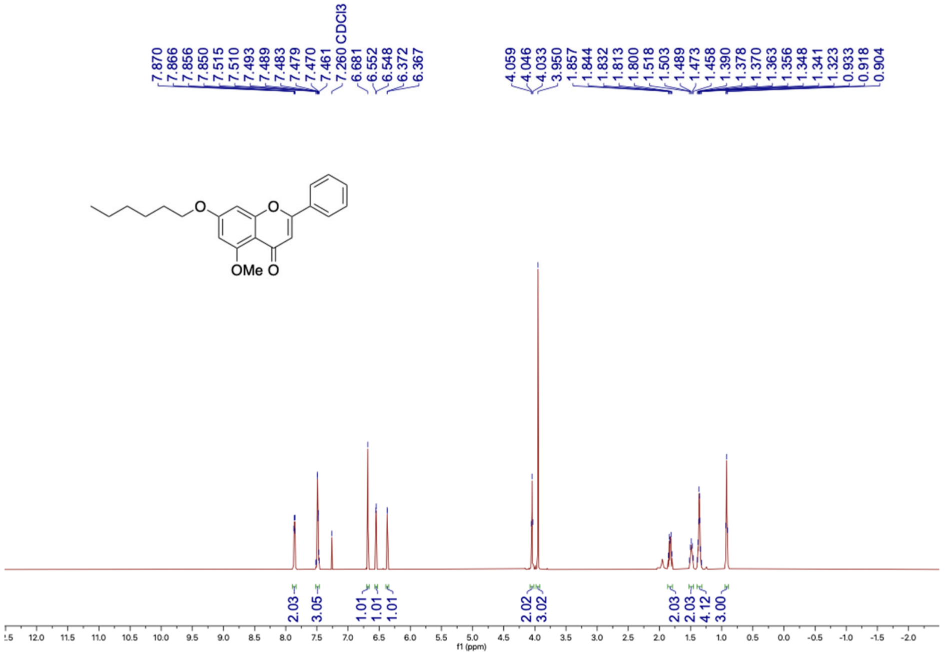

Ljxuh#V581#K#QPU#vshfwuxp#ri#I43#

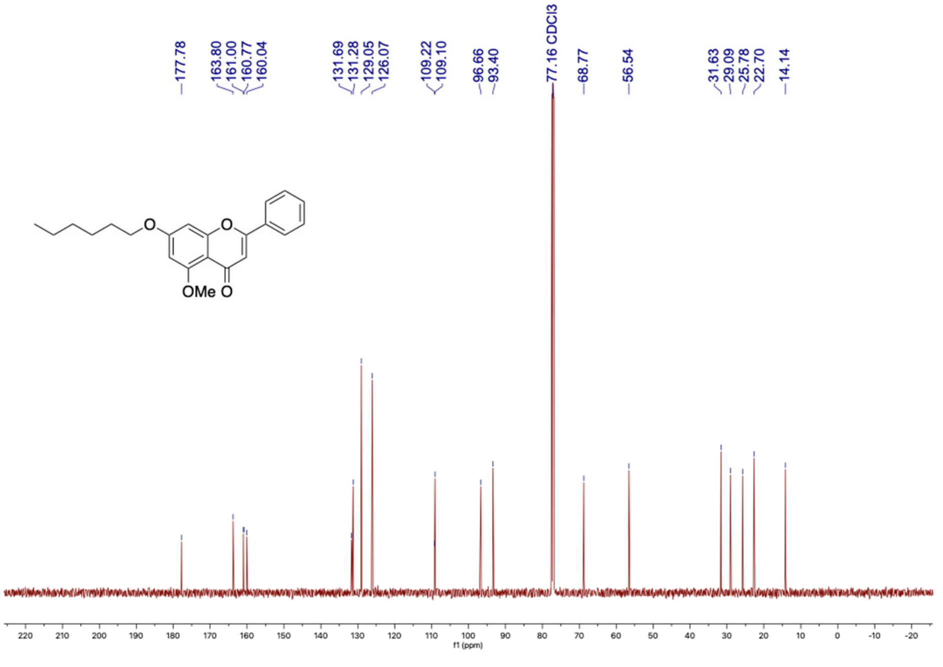

Ljxuh#V591#F#QPU#vshfwuxp#ri#I43#

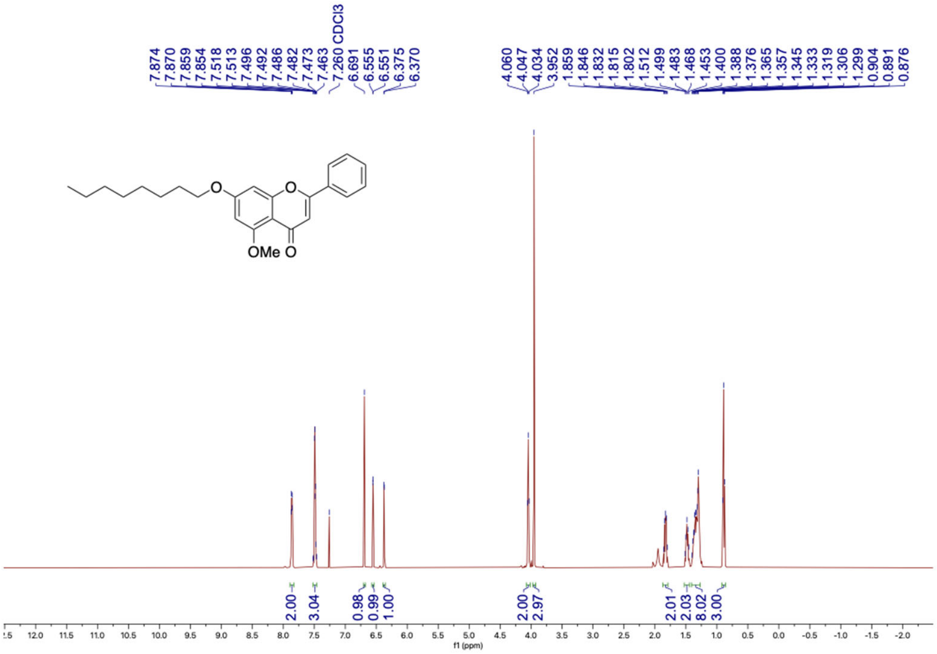

#

Ljxuh#V5:1#4K#QPU#vshfwuxp#ri#I44#

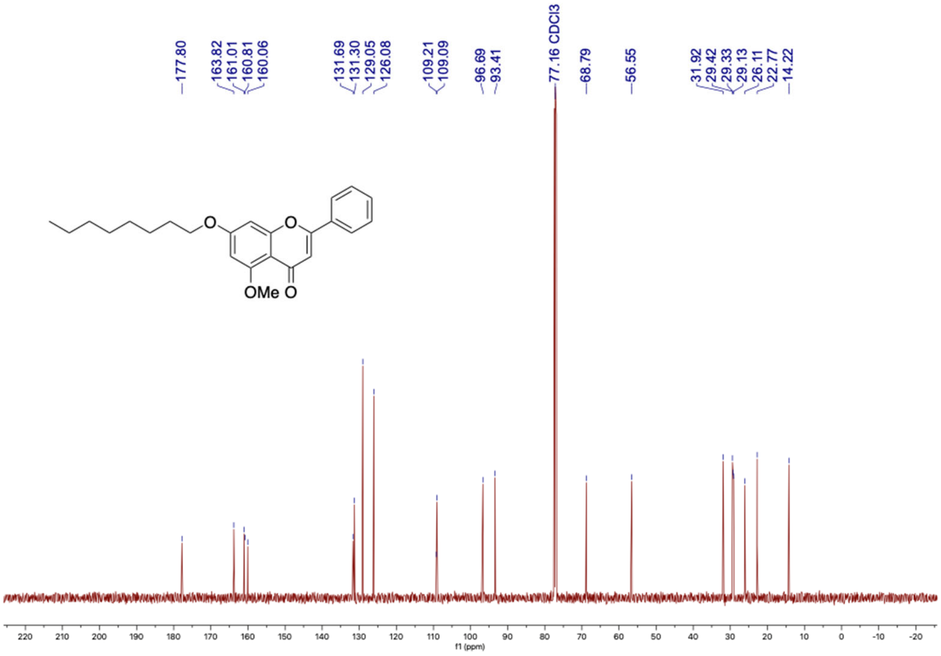

#

Ljxuh#V5;1#46F#QPU#vshfwuxp#ri#I44#

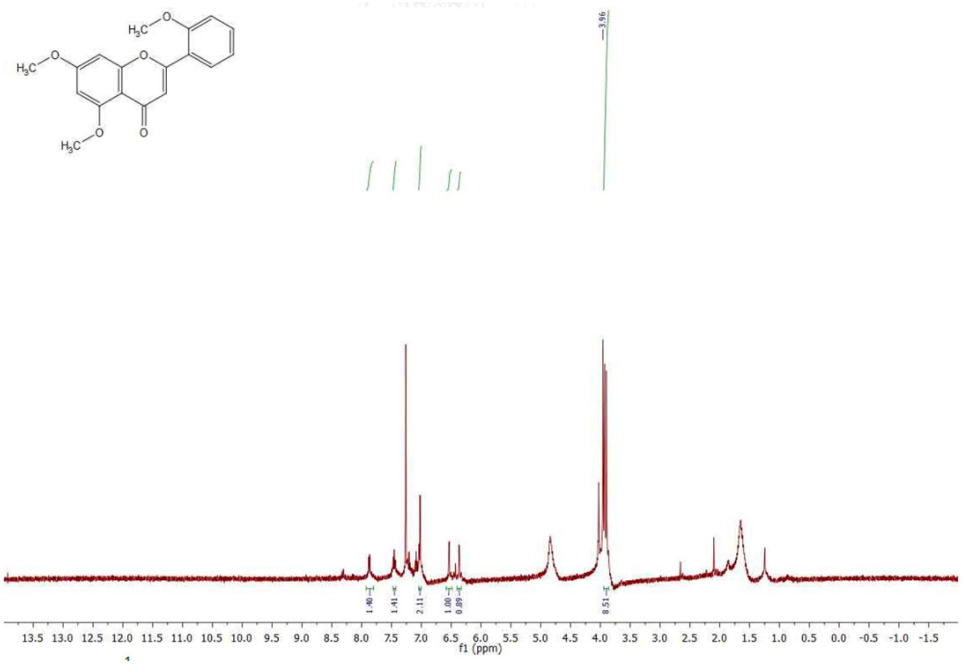

#

Iljxuh#V5<1#^{4}K#QPU#vshfwuxp#ri#I45#

#

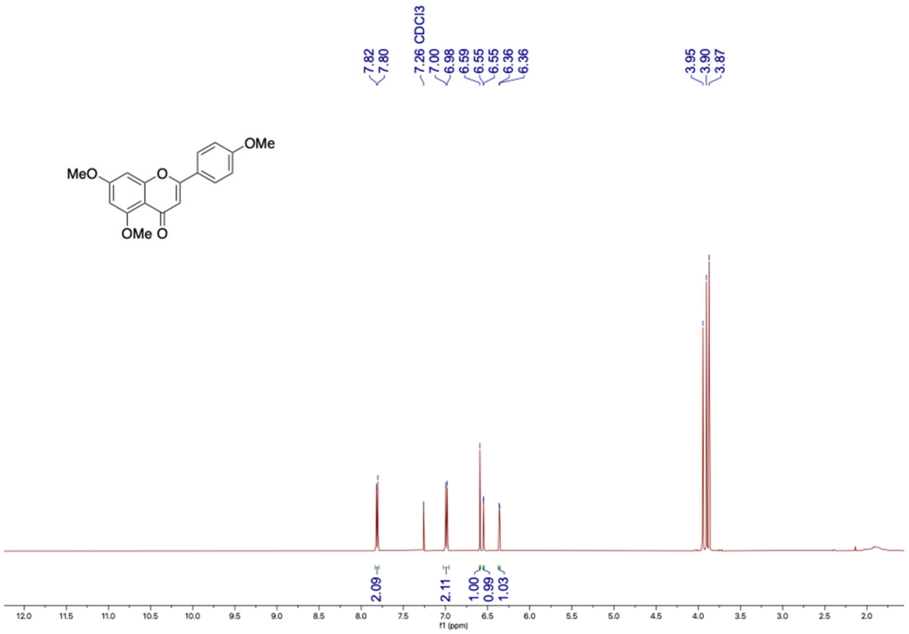

#

Iljxuh#V631#^{4}K#QPU#vshfwuxp#ri#I46#

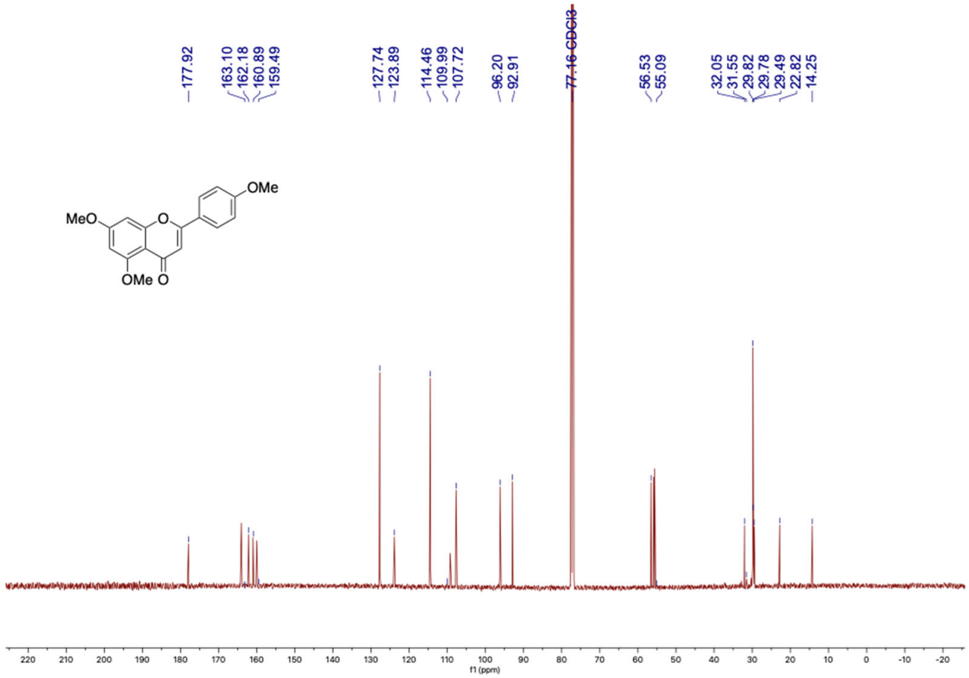

#

Iljxuh#V641#^{46}F#QPU#vshfwuxp#ri#I46#

#

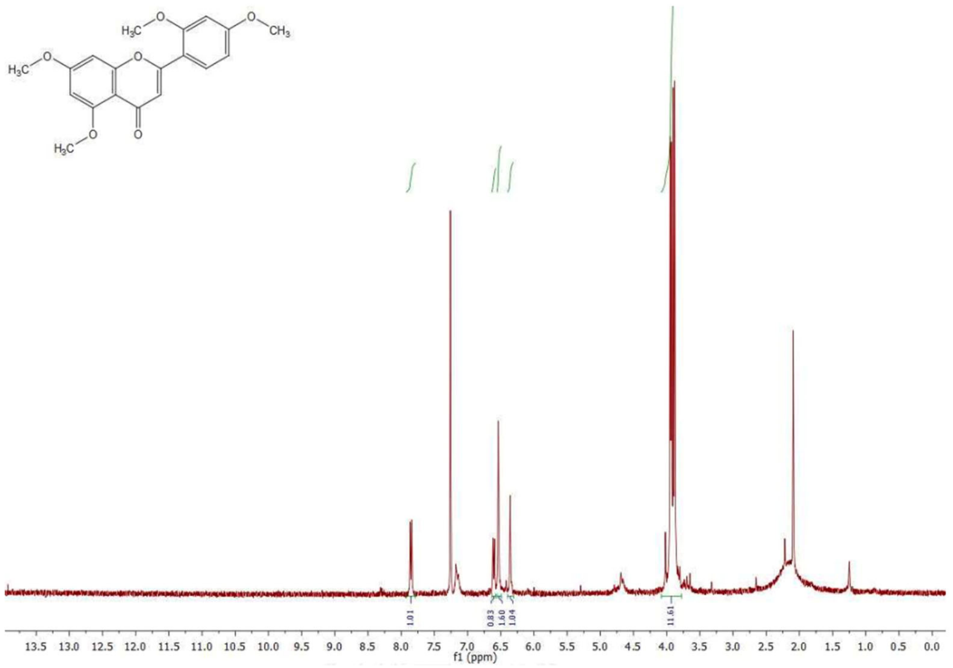

#

Iljxuh#V651#^{4}K#QPU#vshfwuxp#ri#I47#

#

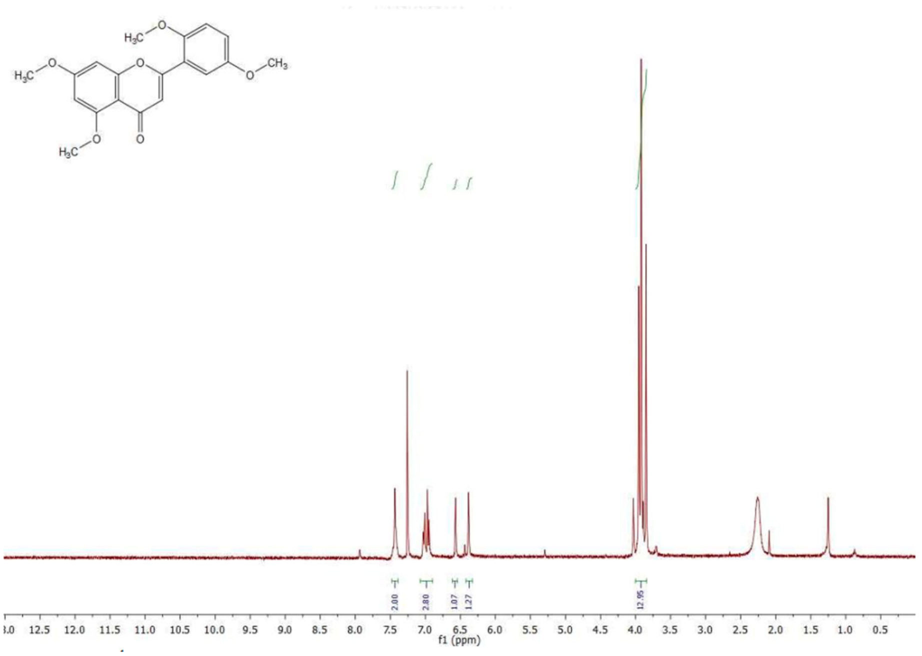

#

Ijxuh#V66#4K#QPU#vshfwuxp#ri#I48#

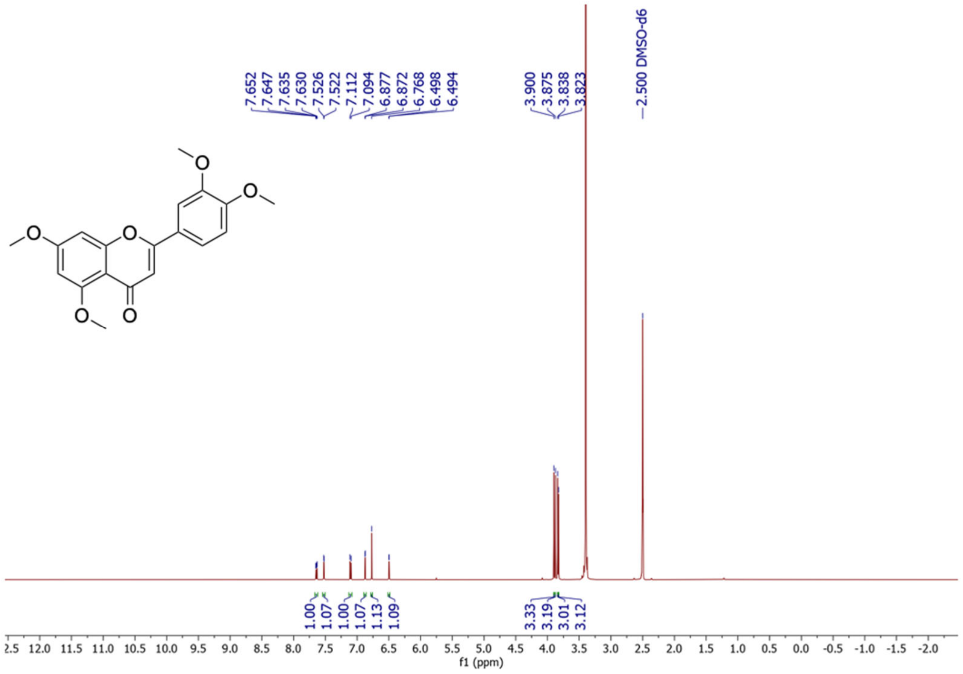

#

Ijxuh#V67#4K#QPU#vshfwuxp#ri#I49#

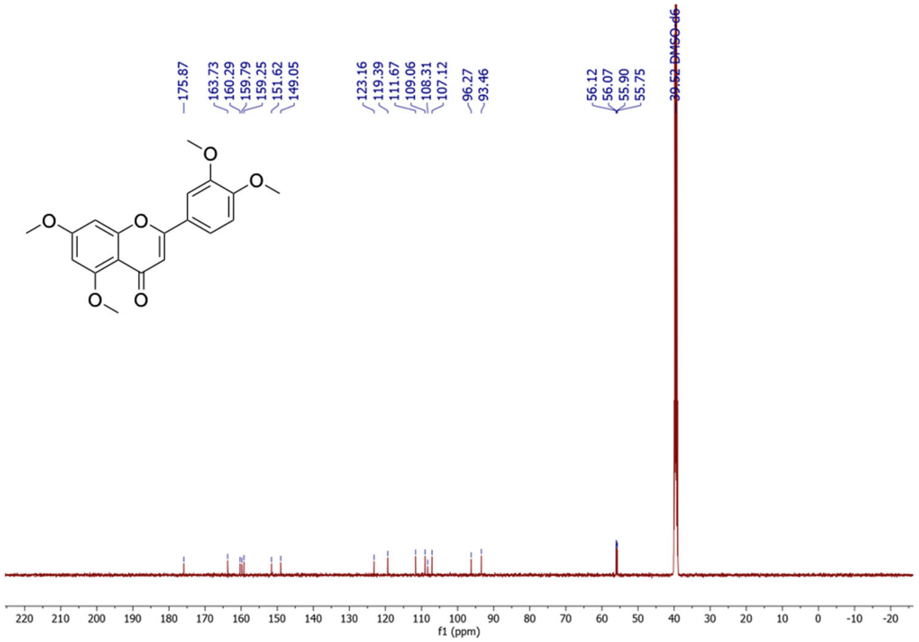

#

Ijxuh#V681#^{46}F#QPU#vshfwuxp#ri#I49#

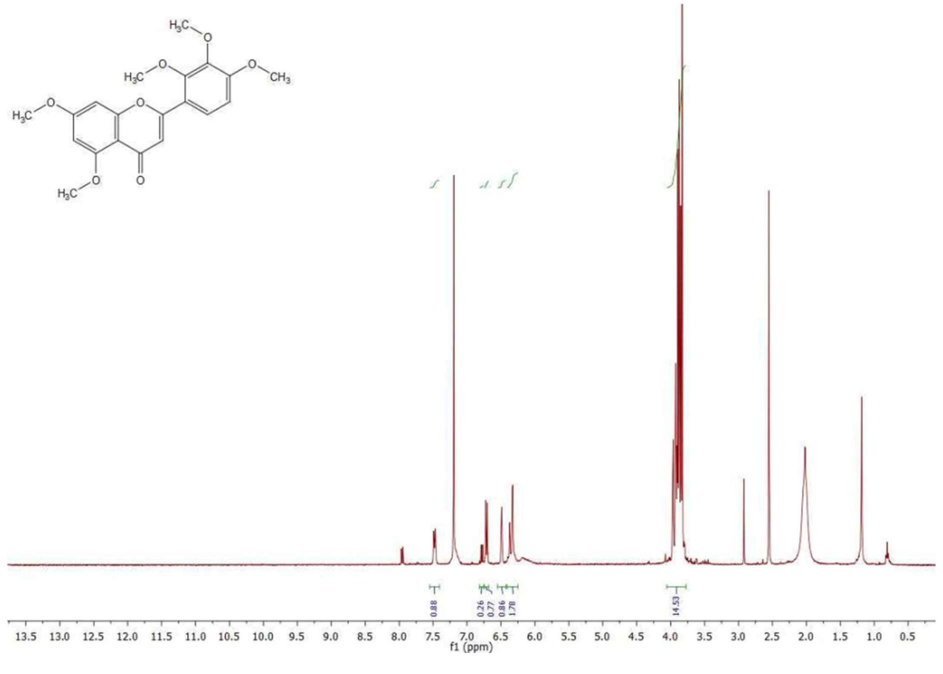

#

Ijxuh#V691#K#QPU#vshfwuxp#ri#I4:#

#

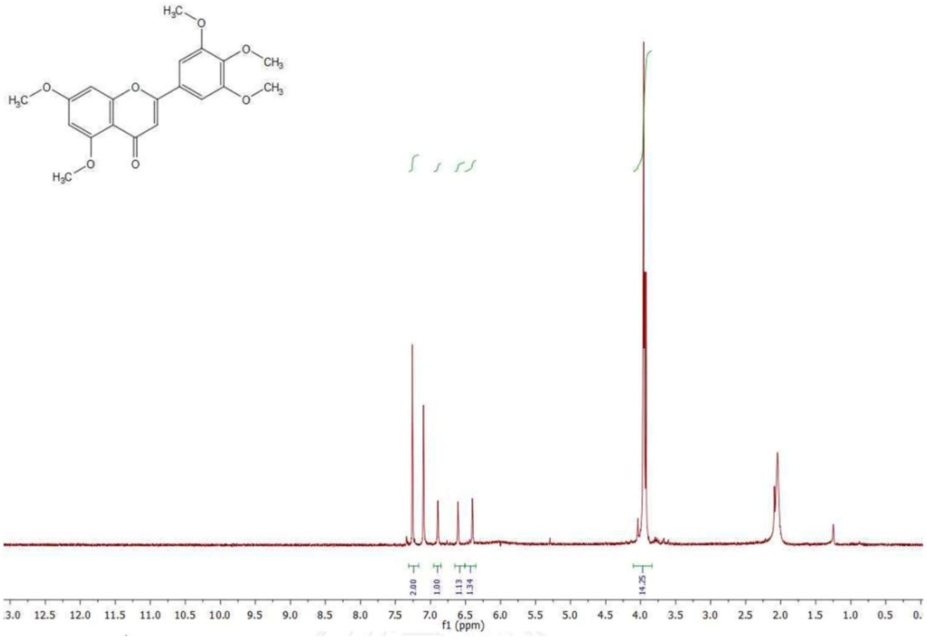

#

Ljxuh#V6:1#K#QPU#vshfwuxp#ri#4;#

#

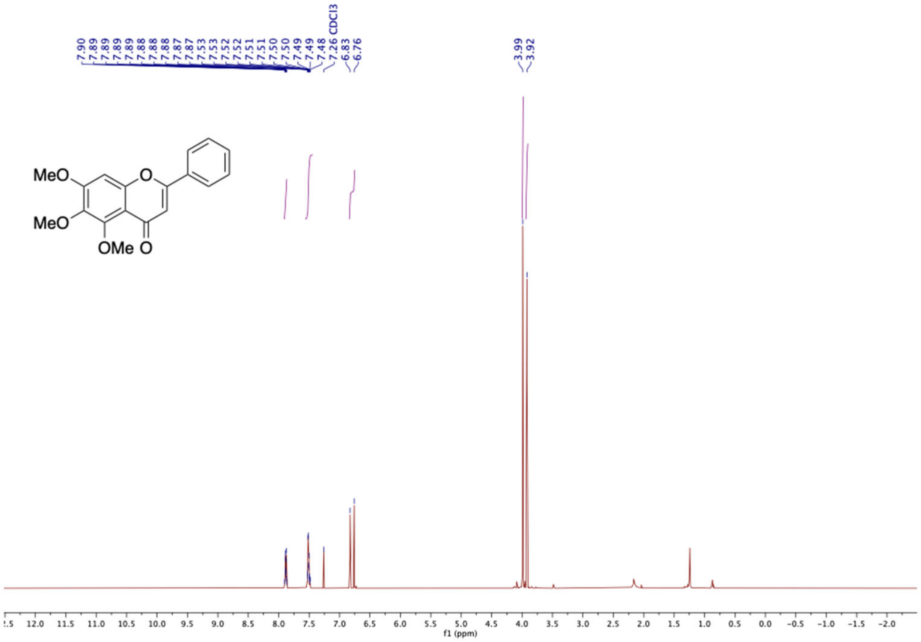

#

Ljxuh#V6;1#K#QPU#vshfwuxp#ri#4<#

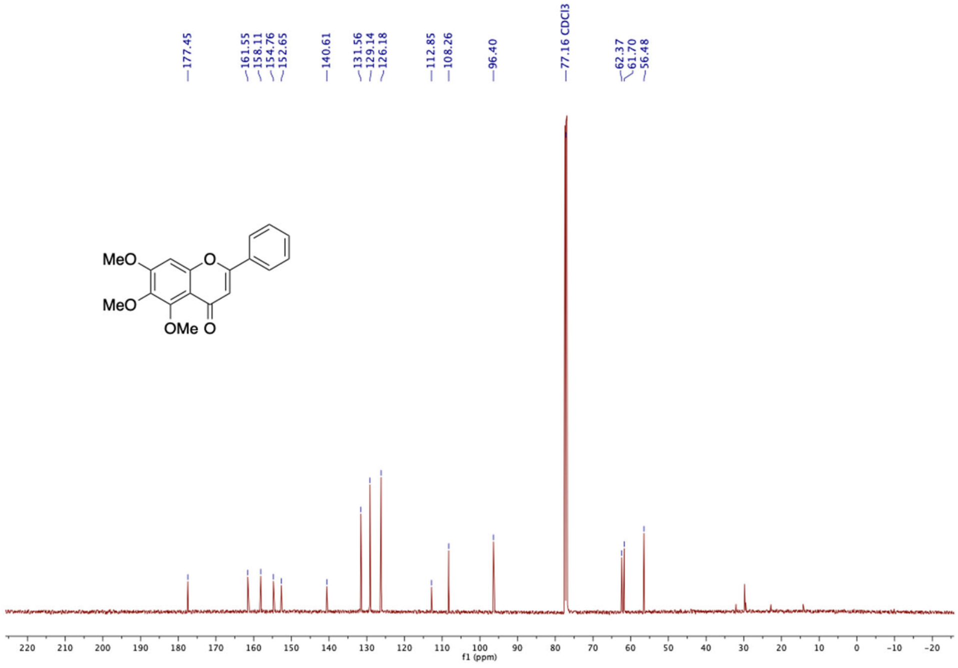

Ljxuh#V6<1#46F#QPU#vshfwuxp#ri#I4<#

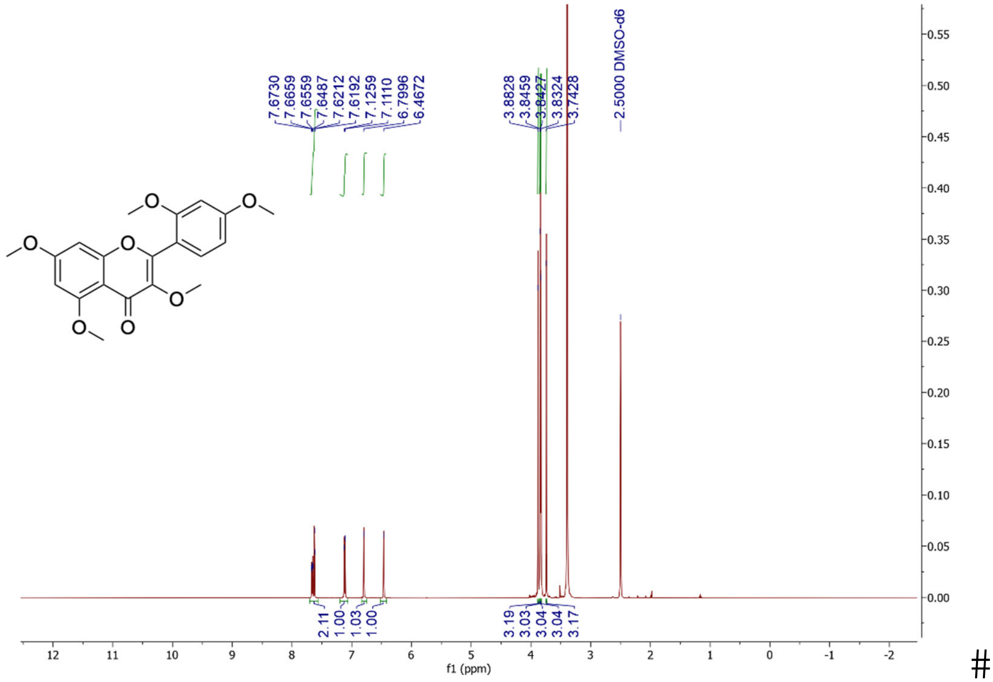

Ljxuh#V731#4K#QPU#vshfwuxp#ri#I53#

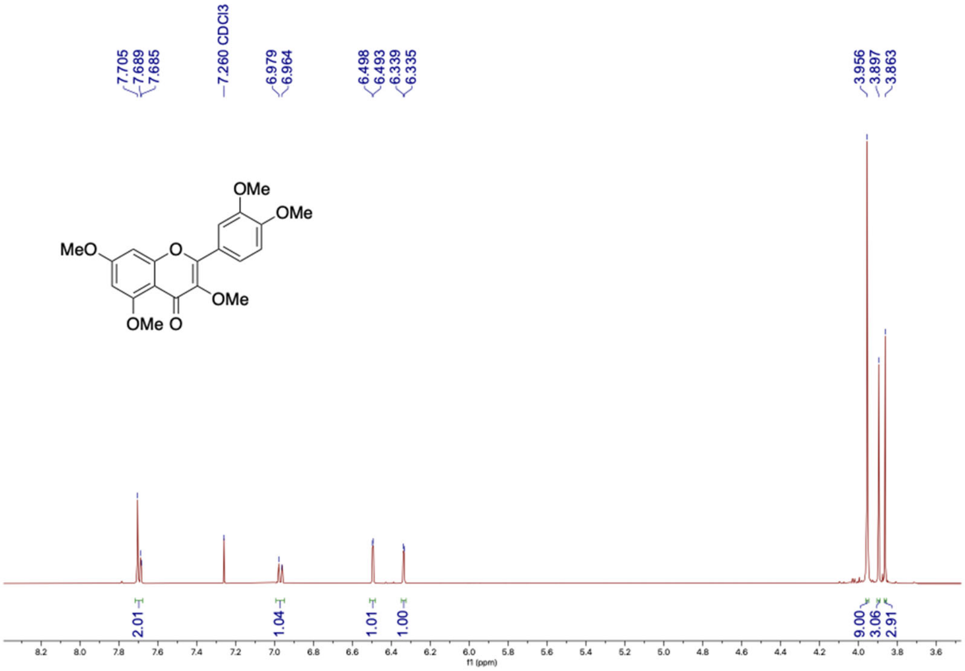

#

Ljxuh#V741#4K#QPU#vshfwuxp#ri#I54#

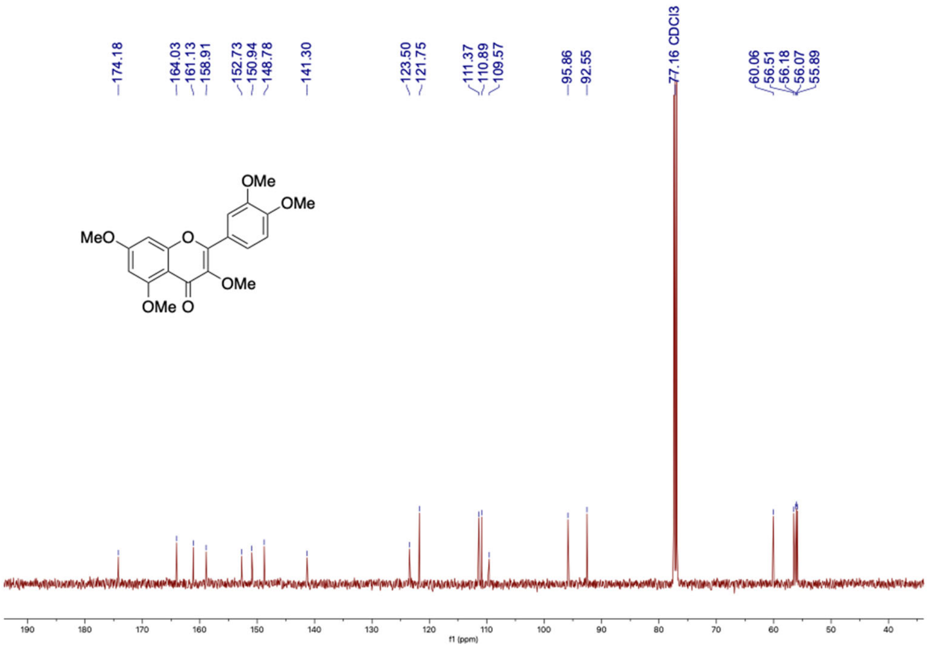

#

Ljxuh#V751#46F#QPU#vshfwuxp#ri#I54#

#

#

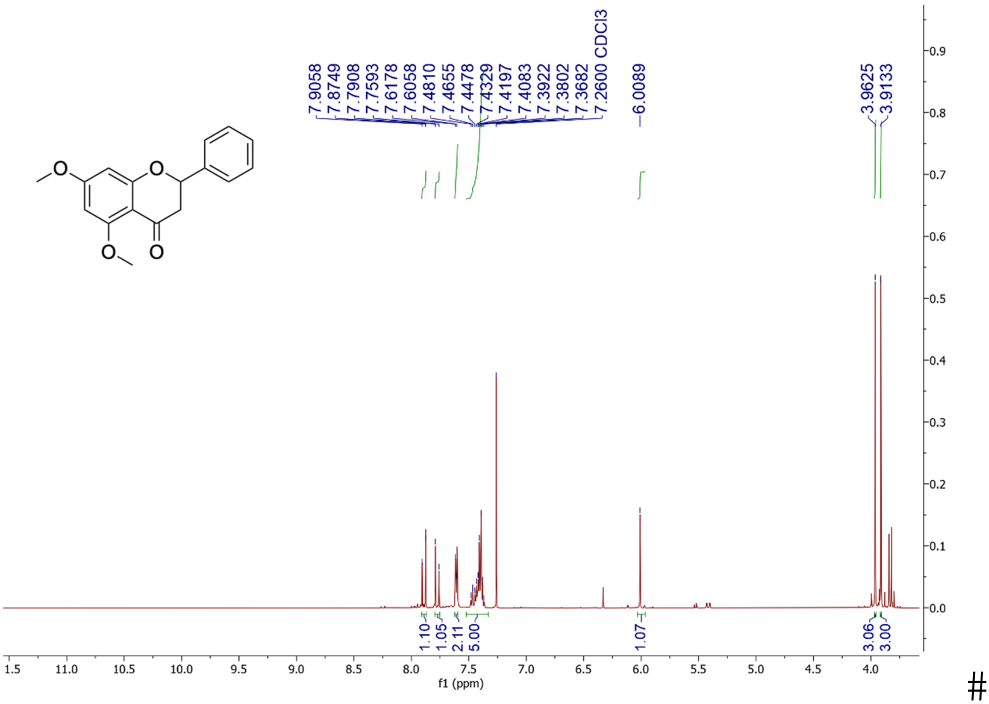

#

Iljxuh#V761#4K#QPU#vshfwuxp#ri#I55#

#

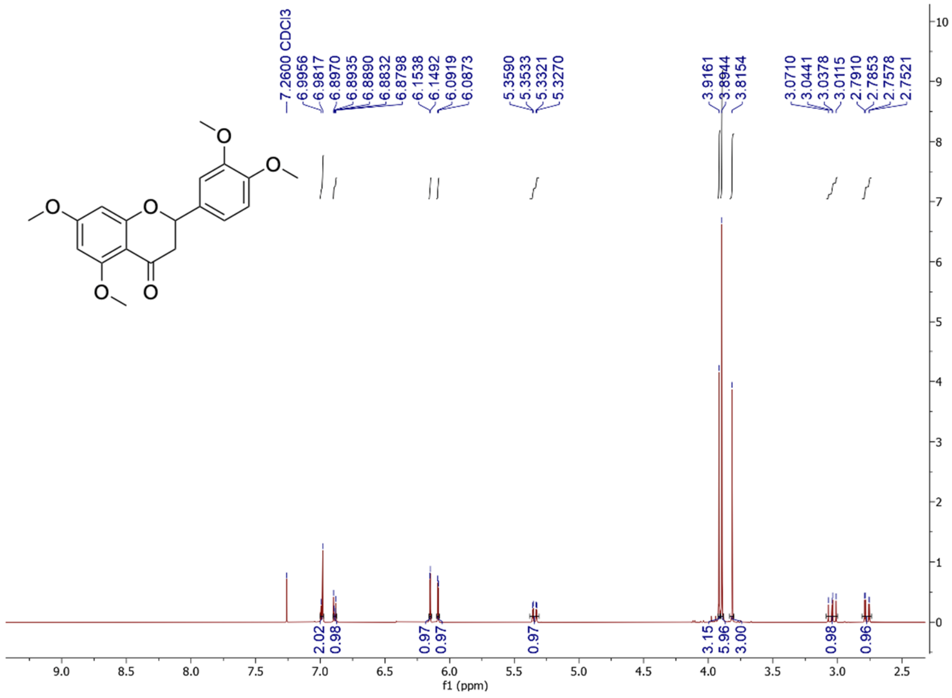

#

Iljxuh#V771#4K#QPU#vshfwuxp#ri#I56#

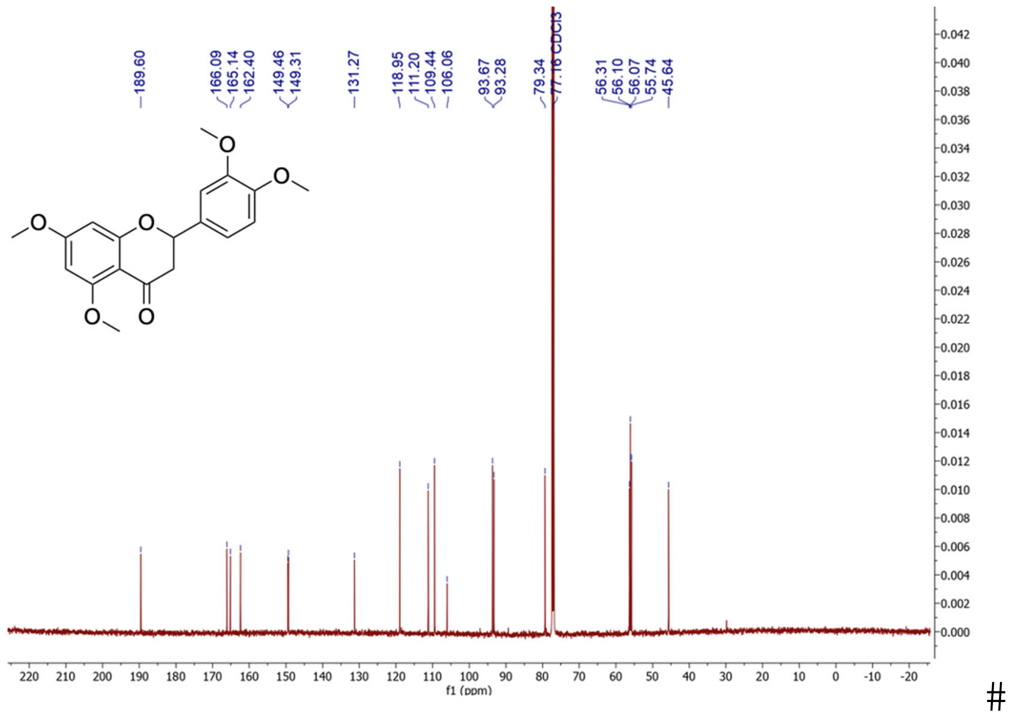

#

Ijxuh#781#46F#QPU#vshfwuxp#ri#56#

#
